# MicotoXilico: An Interactive Database to Predict Mutagenicity, Genotoxicity, and Carcinogenicity of Mycotoxins

**DOI:** 10.3390/toxins15060355

**Published:** 2023-05-24

**Authors:** Josefa Tolosa, Eva Serrano Candelas, José Luis Vallés Pardo, Addel Goya, Salvador Moncho, Rafael Gozalbes, Martina Palomino Schätzlein

**Affiliations:** 1 Laboratory of Food Chemistry and Toxicology, Faculty of Pharmacy, University of Valencia, Av. Vicent Andrés Estellés, Burjasot, 46100 Valencia, Spain; 2 ProtoQSAR S.L., CEEI-Technology Park of Valencia, Av. Benjamín Franklin, 12, 46980 Paterna, Spain; eserrano@protoqsar.com (E.S.C.); jlvalles@protoqsar.com (J.L.V.P.); agoya@protoqsar.com (A.G.); smoncho@protoqsar.com (S.M.); rgozalbes@protoqsar.com (R.G.); 3 Moldrug AI Systems S.L., Olimpia Arozena Torres, 45, 46018 Valencia, Spain

**Keywords:** mycotoxins, QSAR, mutagenicity, genotoxicity, carcinogenicity

## Abstract

Mycotoxins are secondary metabolites produced by certain filamentous fungi. They are common contaminants found in a wide variety of food matrices, thus representing a threat to public health, as they can be carcinogenic, mutagenic, or teratogenic, among other toxic effects. Several hundreds of mycotoxins have been reported, but only a few of them are regulated, due to the lack of data regarding their toxicity and mechanisms of action. Thus, a more comprehensive evaluation of the toxicity of mycotoxins found in foodstuffs is required. In silico toxicology approaches, such as Quantitative Structure–Activity Relationship (QSAR) models, can be used to rapidly assess chemical hazards by predicting different toxicological endpoints. In this work, for the first time, a comprehensive database containing 4360 mycotoxins classified in 170 categories was constructed. Then, specific robust QSAR models for the prediction of mutagenicity, genotoxicity, and carcinogenicity were generated, showing good accuracy, precision, sensitivity, and specificity. It must be highlighted that the developed QSAR models are compliant with the OECD regulatory criteria, and they can be used for regulatory purposes. Finally, all data were integrated into a web server that allows the exploration of the mycotoxin database and toxicity prediction. In conclusion, the developed tool is a valuable resource for scientists, industry, and regulatory agencies to screen the mutagenicity, genotoxicity, and carcinogenicity of non-regulated mycotoxins.

## 1. Introduction

Mycotoxins are common contaminants present in several human food products and animal feed. They are produced during the secondary metabolism of different filamentous fungi (molds), being the most common mycotoxin-producing fungal species *Aspergillus*, *Fusarium*, *Penicillium*, *Claviceps*, and *Alternaria* [1]. These genera are responsible for the production of an important variety of mycotoxins, which also include the main mycotoxins reported, such as aflatoxins, trichothecenes, fumonisins, ochratoxins, patulin, and zearalenone [2]. Some differences can be observed between strains: while *Fusarium* species usually infect growing crops in the field, *Aspergillus* and *Penicillium* species frequently grow on foods and feeds during the storage stage [3]. As a result, there is a wide range of foodstuffs susceptible to contamination by mycotoxins, including cereals, nuts, pasta, fruits, coffee, and by-products of animal origin [4]. Thus, mycotoxins can enter the food chain directly from plant-based food components contaminated with mycotoxins or due to the consumption of animal-derived products from animals fed contaminated feedstuffs, due to the carry-over of mycotoxins to animal-derived products such as milk, meat, and eggs [3]. Even when excellent agronomic, storage, and processing techniques are used, mycotoxin contamination of food and feed is still an unavoidable and unpredictable hazard, creating a challenging risk for food safety. Therefore, mycotoxin occurrence in foodstuffs is an actual problem; indeed, mycotoxins are the main hazard reported in European border rejection notifications by the Rapid Alert System for Food and Feed (RASFF) [3].

Moreover, it is worth highlighting that the pattern of mycotoxin production by several fungi in different geographical distribution is being affected by climate change, generating increasing concern [5]. For instance, the rises in temperature and rainfall in some geographical regions may result in more favorable environmental conditions for *Fusarium*, as in Europe. On the other hand, longer and more frequent droughts may encourage *Aspergillus flavus* to produce aflatoxins under both pre-harvest and post-harvest settings. In addition, recent investigations have demonstrated that the growth of mycotoxin-producing fungi can be stimulated even by a slight elevation in CO_2_ levels [6,7]. Thus, the changing climatic conditions we are facing nowadays could change mycotoxin production and distribution worldwide.

Consumption of mycotoxin-contaminated food or feed can induce acute or chronic toxicity in humans and animals, with chronic effects being the most prevalent, due to prolonged exposure to lower concentrations. As a result, regulations concerning mycotoxins have been implemented in many countries to safeguard consumers from the harmful effects of these compounds [3,8]. Nevertheless, as regulations are primarily based on known toxic effects, maximal allowed limits, or tolerable daily intakes (TDI) were determined only for a few mycotoxins, as there are many mycotoxins for which no experimental data exist [9]. 

Therefore, additional risk assessment surveys on non-regulated mycotoxins are urgently required. However, the performance of traditional in vivo assays on thousands of different compounds would be extremely expensive and unethical. In this context, the application of alternative methods, such as in silico strategies could be extraordinarily useful. Indeed, several guidance documents have been drafted to improve standardization, harmonization, and uptake of in silico methods by regulatory authorities including the EFSA (European Food Safety Authority) and the ECHA (European Chemicals Agency) [10,11]. In this sense, the Commission Regulation No. 1907/2006 called REACH (Registration, Evaluation and Authorisation of Chemicals) (http://ecb.jrc.it/reach/reachlegislation/) (accessed on 23 January 2023) foresees the use of in silico methods such as (Quantitative) Structure–Activity Relationship ([Q]SAR) models when the same level of information can be obtained by means other than in vivo testing [10,11,12]. More concretely, information relating to the genotoxic potential of chemicals by using in silico prediction approaches has become an important source, as recommended by the REACH regulation as well as the ICH M7 guideline for the assessment and control of DNA reactive or mutagenic impurities [12,13,14,15]. 

Among the toxic effects that can be caused by mycotoxins, the induction of genetic alterations is an important matter of concern [14,16,17,18], as several mycotoxins and some of their metabolites have been described as genotoxic compounds, including aflatoxins, ochratoxins, citrinin, and HT-2 and T-2 toxins [19]. However, scarce information has been reported regarding the capability of other mycotoxins to cause these adverse effects. 

A genotoxic compound can induce mutations (mutagenicity) and/or cause the generation of tumors (carcinogenicity). To characterize these properties, an in silico toxicology (IST) protocol template [20] as well as a protocol for genetic toxicology (the GIST protocol) [14] have been designed and developed by an international consortium comprising several industry, academia, and government agencies. Therefore, in the present study, in vitro tests recommended for the mutagenicity and genotoxicity endpoints by the GIST have been taken into account to search experimental data for QSAR model building. To define the mutagenicity, the in vitro bacterial reverse mutation assay (commonly referred to as the Ames test) provides robust and high-quality data, which have been previously used to develop QSAR models with a good performance in predicting mutagenic activity. On the other hand, for genotoxicity assessment, data on in vitro micronucleus (MN) assay have been widely used, while carcinogenicity is evaluated by detecting tumor generation in in vivo models. 

The aim of the present study was the development of specific QSAR models to predict genotoxicity, mutagenicity, and carcinogenicity of a wide range of mycotoxins. To this end, we build, for the first time, a comprehensive database including almost 4400 different mycotoxins, clustered in different categories according to their chemical structure. We then overlapped this list with different databases of genotoxicity, mutagenicity, and carcinogenicity to obtain experimental data based on the Ames test for mutagenicity, in vitro and in vivo MN assay for genotoxicity, and data from in vivo models for carcinogenicity. These data were then applied for the building and validation of scientifically valid and robust QSAR models that predicted the endpoints on a test set of mycotoxins with a high accuracy, sensitivity, and selectivity. Finally, the mycotoxins database together with the predicted toxicity values was integrated in a new, open access web server that can be explored interactively. 

## 2. Results

### 2.1. Mycotoxin Database and Clustering

Our search resulted in a data set of 4360 mycotoxins identified by their name, isomeric SMILES (Simplified Molecular Input Line Entry System) and PubChem CID. In addition, we grouped the mycotoxins according to their chemical structure in 170 different families. To our knowledge, this is the first time that such a comprehensive database of mycotoxins has been published, as previous works only made reference to several hundred mycotoxins [21,22,23,24,25]. To provide an easy access to the database, we have created a web application, MicotoXilico, that allows easy exploration of the data (https://chemopredictionsuite.com/MicotoXilico, accessed on 20 May 2023). 

**Figure 1:**
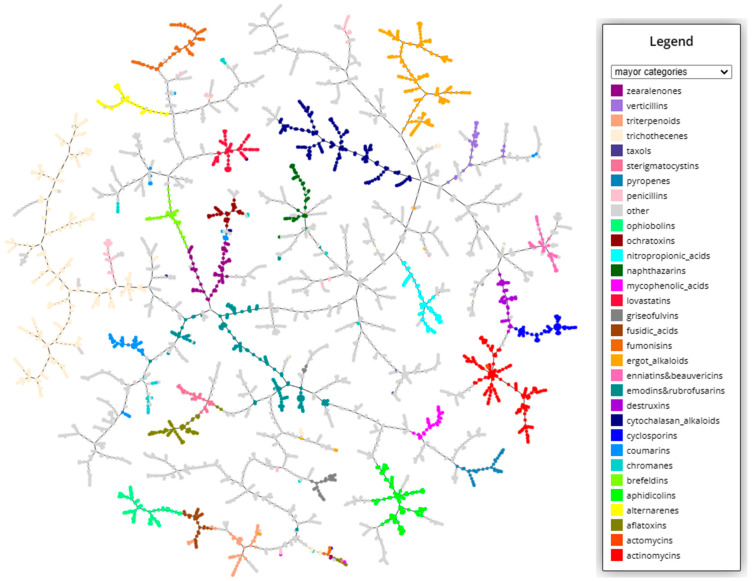
TMAP clustering graph of the database containing 4360 mycotoxins. The most important categories are labelled in different colors. For an interactive exploration of all categories, please visit MicotoXilico (https://chemopredictionsuite.com/MicotoXilico, accessed on 20 May 2023).

In order to better visualize the structural diversity of the mycotoxins, a clustering based on k-nearest neighbor approach from structural fingerprints was performed (https://tmap.gdb.tools/ accessed on 20 May 2023). The resulting graphs can be explored interactively at the MicotoXilico web. In Figure 1, a clustering plot indicating the major categories of mycotoxins is depicted. In the graph, the number of linkages between compounds is proportional to their chemical similarity, meaning that similar mycotoxins will be connected through short pathways. 

As we see, myctoxins have a very high structural variability, representing almost the full range of natural products. They include alkaloids, terpenoids, peptids, fatty acids, lactones, nucleotids, phenols, and anthraquinones, among others. Until now, even if there were some known categories such as zerealones, ergot alkaloids, or trichothecenes, there exists no general systematic classification of mycotoxins. In our classification, we have maintained the groups based on specific structural motives, including between 5 and 50 different compounds. An overview of the categories and the number of compound in each category can be found in the Appendix A (Table A1). In some cases, when there was a very high similarity between structures, we have created unified groups, such enniatins and beauvericins, emodins and rubrofusarins, asperlins and asperlactones, fusarins and fusariens, or usnic acids and ustins. In other cases, the structural similarity was not big enough to unify categories, but it was still remarkable, for instance, between sphingofungins and fumonisins, alternariolides and enniatins, roquefortines and ergot alkaloids, or brefeldins and zearealones. These similarities can be appreciated in the graphical clustering visualization.

Some of the larger, more traditional categories have a higher structural variability because they include several subgroups of compounds. For instance, cytochalasan alcaloids can be subclassified into chaetoglobosins, daldinins, and chalasins. In particular, trichothecenes, the most abundant category, with more than 350 compounds, has a high number of subcategories, as can be explored in the web server (https://chemopredictionsuite.com/MicotoXilico, accessed on 20 May 2023).

Some compounds could not be associated with a specific structural category, and they have therefore been classified into more general groups comprising alkaloids, terpenoids, amino acid derivatives, peptides, clyclic peptides, nucleotides, anthraquinones, benzoquinones, naphthalenes, phenols, phtalates, furans, lactones, thiazolidines, and fatty acid-like compounds. Furthermore, 79 compounds could not been associated with any category and were labelled as “not classified”.

### 2.2. QSAR Models Building

Once we had generated a comprehensive database of mycotoxins, we wanted to optimize several machine learning models to predict the genotoxicity, mutagenicity, and carcinogenicity of these compounds. The tests chosen for the characterization of each type of toxicity follow the recommended endpoint protocols of the OECD [26]. For data searching, we overlapped our ensemble of mycotoxins with different databases of mutagenicity, genotoxicity, and carcinogenicity to obtain experimental data based on the Ames test for mutagenicity, in vitro and in vivo MN assay for genotoxicity, and data from in vivo models for carcinogenicity. As expected, experimental data could only be found for a relatively low number of compounds (350–100 compounds, depending on the endpoint).

These data were then used for the building and validation of robust QSAR models to predict the four endpoints. For model building, we followed the protocols described in the materials and methods section that meet the requirement of the five principles of the OECD for QSAR model building in a regulatory context [27].

**Figure 2:**
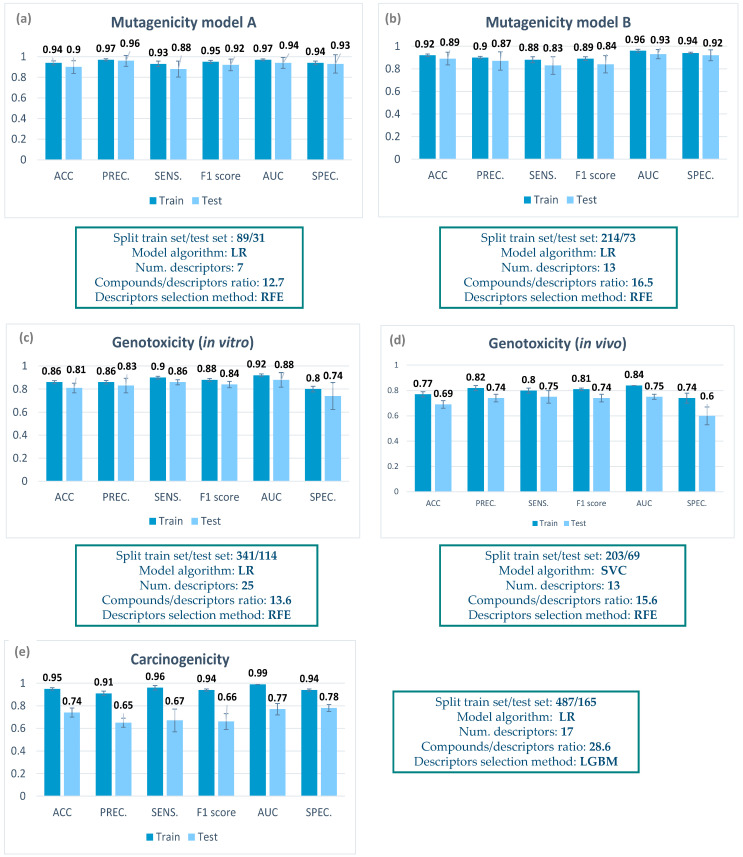
Metrics for QSAR models of (**a**) mutagenicity model A, (**b**) mutagenicity model B, (**c**) in vitro genotoxicity, (**d**) in vivo genotoxicity, and (**e**) carcinogenicity. ACC: accuracy; PREC: precision; SENS: sensitivity; AUC: area under the curve; SPEC: specificity; LGBM: Light Gradient Boosting Machine Classifier; LR: logistic regression; RFE: recursive feature elimination; SVC: Support Vector Machine Classifier (SVC).

A summary of the characteristics of the final classification models can be found in Figure 2, including the number of compounds in the training and test set, the descriptor selection method, the number of descriptors, the compound/descriptor ratio, and the model algorithm. In the Appendix A, a list with the molecular descriptors selected for each model can be found (Table A2, Table A3, Table A4, Table A5 and Table A6). It is worth mentioning that the descriptors were selected from a panel of more than 4000, including 2D and 3D descriptors, in order to obtain the best chemical description of the complex mycotoxin structure. 

For mutagenicity, models based only on mycotoxin data could be build, as data for up to 365 compounds could be retrieved, with a balanced proportion between mutagenic and non-mutagenic compounds. Two mutagenicity QSAR models (model A and model B) were generated applying two different data selection criteria (for details, see Section 5.3). In both cases, all the parameters included in the metrics were higher than 0.8, thus showing a good performance. Both models were able to correctly predict the mutagenicity of almost 90% of compounds in the internal cross validation.

**Figure 3:**
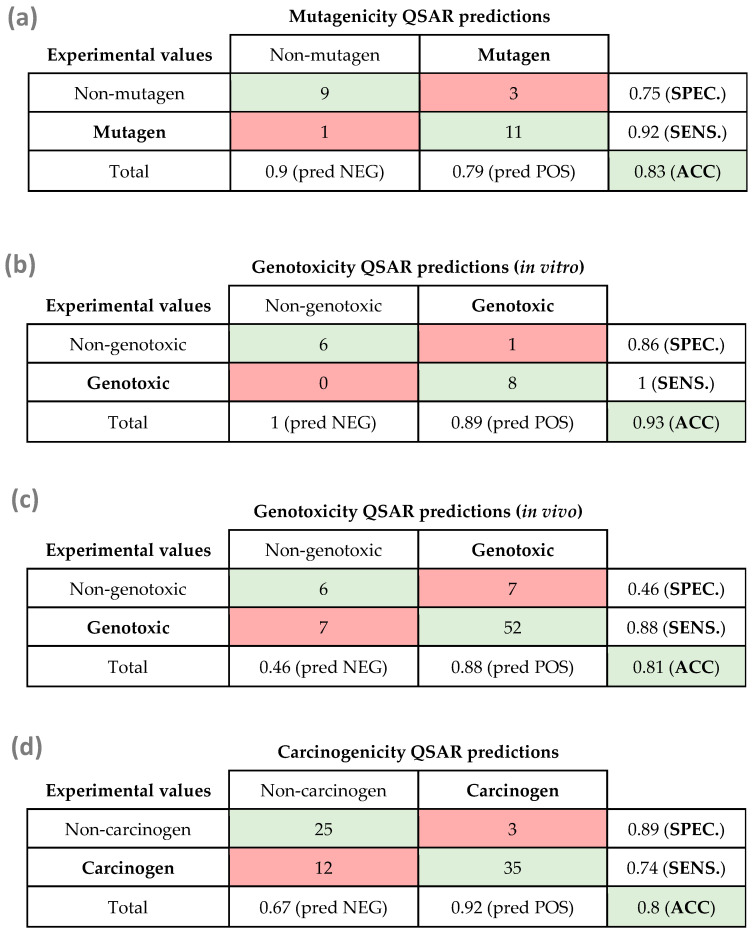
Matrix confusion for the external validation of (**a**) the mutagenicity QSAR model A applied to 24 mycotoxins, (**b**) the in vitro genotoxicity QSAR model applied to 15 mycotoxins, (**c**) the in vivo genotoxicity QSAR model applied to 72 mycotoxins, and (**d**) the carcinogenicity QSAR model applied to 75 mycotoxins. SPEC: specificity; SENS: sensitivity; ACC: accuracy; pred NEG: predicted negatives; pred POS: predicted positives.

For genotoxicity, data retrieval was much more complicated, as very few data from non-genotoxic mycotoxins could be detected. Therefore, we decided to build mixed models including mycotoxins and other organic compounds (for details, see Section 5.4). Regarding the in vitro genotoxicity QSAR model, information on the in vitro MN assay from 455 compounds was considered (Figure 2). For a more comprehensive analysis of genotoxicity, we decided to also include an in vivo genotoxicity model. In this case, the ProtoPRED model (https://protopred.protoqsar.com/, accessed on 20 May 2023) based on the in vivo MN assay was applied, built from a training set that included mycotoxins. Regarding the performance of these models, most parameters were higher than 0.8 (Figure 3d). Only the specificity was moderate, as some non-genotoxic compounds were predicted as genotoxic in the internal validation. This result could relate to the fact that only a few data from non-genotoxic mycotoxins could be found.

For carcinogenicity, again we applied the in vivo carcinogenicity QSAR model from ProtoPRED (https://protopred.protoqsar.com/, accessed on 20 May 2023), containing mycotoxins in the training set, as not enough carcinogenicity data were retrieved to build a specific model. The metrics obtained for the carcinogenicity QSAR model showed a good performance on the training set, with all parameters being higher than 0.9. Parameters on the test set showed lower values, especially for precision and sensitivity, but are still close to 0.7, the value recommended for QSAR models by ECHA [28].

When we compare the metrics of the training and test sets in Figure 2, we can see that there are almost no differences for the mutagenicity models, small differences in the in vitro genotoxicity model, and a higher difference in the carcinogenicity and the in vivo genotoxicity. This result is coherent with the fact that the mutagenicity models were built only with mycotoxins, while in the other models, only part of the training set were mycotoxins, meaning that we have more structural differences between the training and test set. 

### 2.3. QSAR Model Application to an External Validation Set

After model building and internal validation, we decided to further confirm the performance of the models by performing an external validation with an independent set of mycotoxins with known experimental data. This allows us to evaluate if the model is truly predictive or if the model has been overfit to the data used for model building. 

Since only the mutagenicity model A is considered valid from a regulatory point of view, we decided to subject only this model to external validation. The list containing the different mycotoxins used for the external validation for each model can be found in Table A7, Table A8, Table A9 and Table A10 of the Appendix A. Figure 3 shows the resulting confusion matrix for all model validations, proving that the experimental data were in general well predicted.

For mutagenicity (Figure 3a), the confusion matrix showed that the model was capable of correctly classifying more than 0.8 of the compounds (0.83 accuracy). The in vitro genotoxicity QSAR model showed the highest accuracy (0.93) within all developed models. All genotoxic compounds were predicted as positive (1.00 of sensitivity), while only one compound out of 7 non-genotoxic mycotoxins was predicted as genotoxic (0.86 of specificity) (Figure 3b), thus improving the specificity of the internal validation of the in vitro model. For the in vivo genotoxicity model, however, although obtaining good values for accuracy (0.81) and sensitivity (0.88), the specificity was again low (0.46), proving that this model was not accurate for the prediction of non-genotoxic compounds. 

The carcinogenicity model was applied to a validation set of 75 mycotoxins, showing an accuracy of 0.81. The 89% of non-carcinogenic mycotoxins were predicted as inactive compounds, while 77% of carcinogenic mycotoxins were predicted as active compounds (Figure 3d).

Thus, even if the models were built with a small data set of experimental data from mycotoxins, they seem to have a good predictive power on the external validation set of mycotoxins. 

In order to see how well our models were adapted to mycotoxins, in comparison with other, more general toxicological QSAR models, we also performed a prediction with the validation data set applying three other reference QSAR tools: VEGA [29], Leadscope, and Case Ultra (the last two being integrated into QSARToolbox) [30]. We predicted the same four endpoints, obtained from the same or a very similar protocol. The metrics of these predictions can be found on Table A11. 

We can observe that, in general, our models provide a better prediction for the selected test mycotoxins. Only for mutagenicity do we obtain a better prediction with Case Ultra; however, six compounds could not be predicted by this model because they were outside the applicability domain. Also, for the other models, the prediction of several mycotoxins could not be performed because they were not in the applicability domain. We have also found that, in some cases, VEGA could not correctly read and normalize structures with aromatic rings, probably due to the aromaticity model that the software uses. These results show that our models are better adapted to complex structures with several aromatic rings, which are typical mycotoxin structures. 

### 2.4. Mutagenicity, Genotoxicity, and Carcinogenicity Prediction of the General Mycotoxin Database

**Figure 4:**
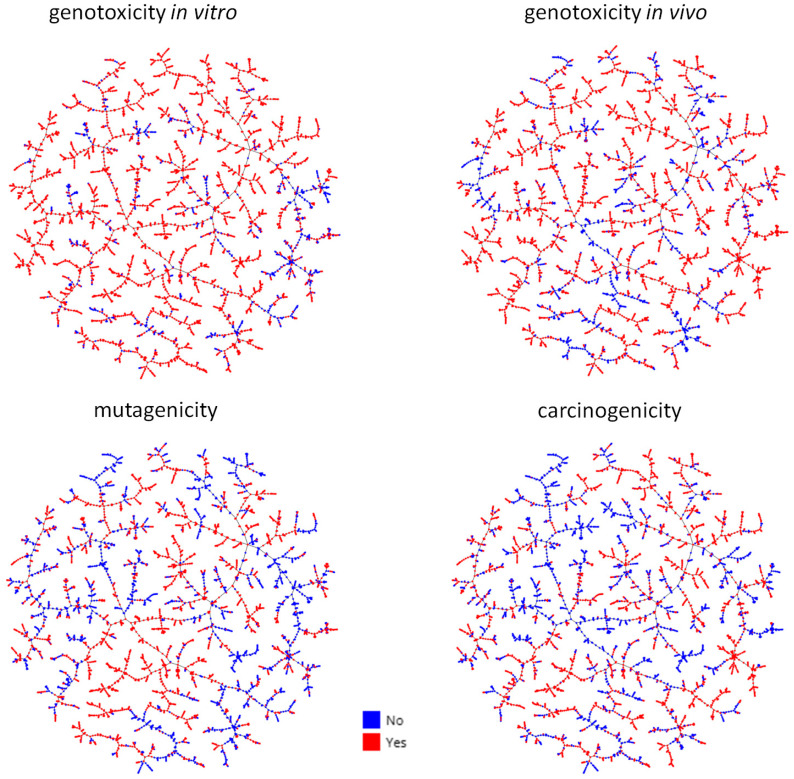
Global overview of genotoxicity, mutagenicity, and carcinogenicity predictions of the whole database of mycotoxins. Clustering was performed with the TMAP package. Blue = non-toxic; red = toxic. For more details, please visit MicotoXilico (https://chemopredictionsuite.com/MicotoXilico accessed on 20 May 2023).

After model building, we wanted to perform a prediction of the genotoxicity, mutagenicity, and carcinogenicity of the whole mycotoxin database described in Section 2.1. A general overview of the results is presented in Figure 4, and detailed predictions can be explored in the MicotoXilico web application (https://chemopredictionsuite.com/MicotoXilico, accessed on 20 May 2023). 

From the figure, we can appreciate that a very high percentage of mycotoxins are predicted to be genotoxic, mutagenic, and/or carcinogenic. This result is not surprising, as the definition of mycotoxin already assumes that we are dealing with toxic compounds. In particular, genotoxicity seems to be a property of most mycotoxins, as 80–90% of these compounds are predicted as genotoxic. However, further studies are required to confirm these results, as the models were built with only a few data from non-genotoxic mycotoxins, and the genotoxicity models only have a moderate specificity. 

We also used the Benigni and Bossa rules for mutagenicity [31], implemented in ProtoICH software (https://protopred.protoqsar.com/, accessed on 20 May 2023) to identify the presence of structural mutagenicity alerts in our database. The number of molecules detected as positive based on structural alerts were significantly less in comparison with the molecules detected as positive based on QSAR models (https://chemopredictionsuite.com/MicotoXilico, accessed on 20 May 2023). On the contrary, when we performed the same comparison with a set of over 6000 general organic compounds, most of the compounds predicted as mutagenic had a mutagenicity alert (data not shown).

**Figure 5:**
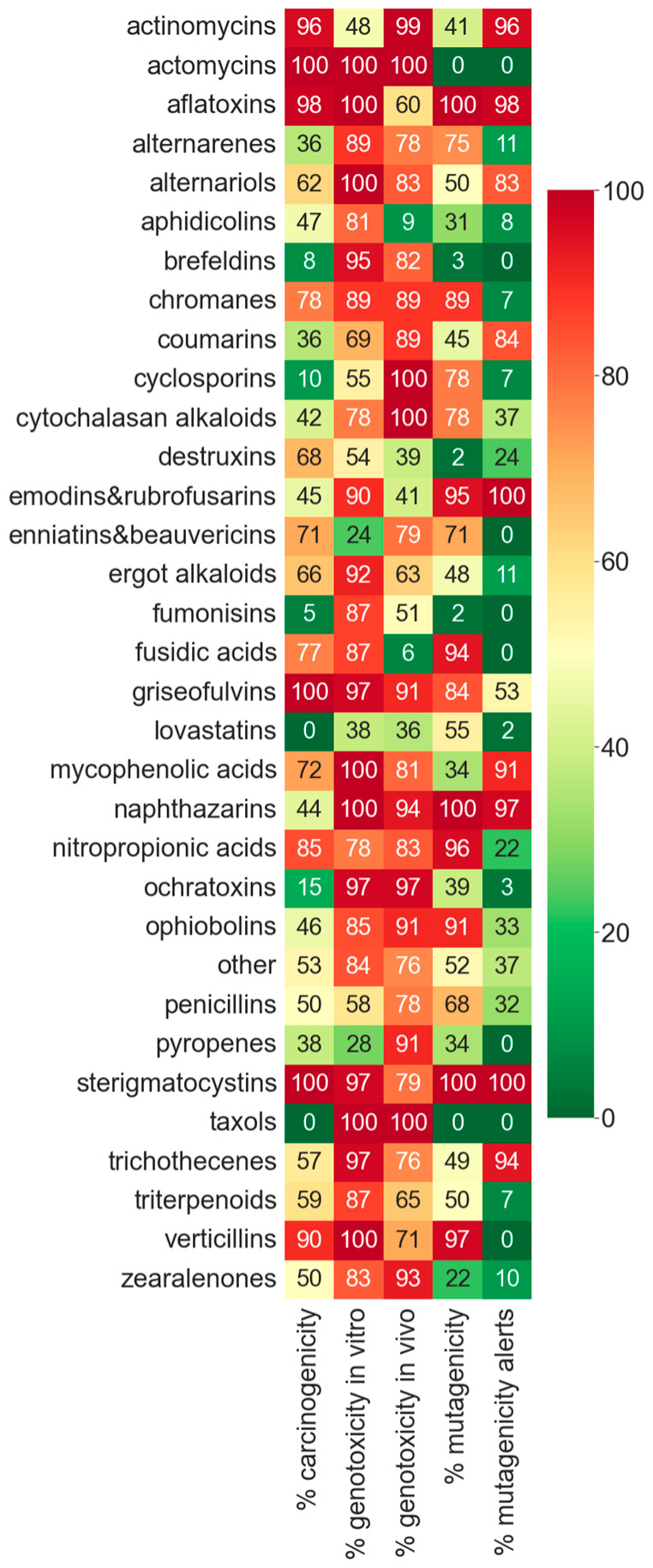
Graphical representation of the percentage of genotoxic, carcinogenic, and mutagenic mycotoxins from the major categories obtained after prediction with the corresponding QSAR models.

In order to compare the toxicity between mycotoxin categories, we represented the percentage of genotoxic, mutagenic, and carcinogenic compounds for the 30 major mycotoxin categories in a heatmap (Figure 5). 

As we can see, there are several categories that are specially concerning, as they have a very high toxicity prediction in mutagenicity, genotoxicity, and carcinogenicity (Figure 5), such as aflatoxins, chromanes, fusidic acids, griseofulvins, nitropropionic acids, sterigmatocystins, and verticillins. Some categories have non-positive predictions in some of the toxicity endpoints (taxols and lovastatins), but there is no type of compound that is negative in all three toxicities. 

In many categories, the mutagenicity alerts correspond to a very high mutagenicity prediction (<80%), such as in aflatoxins, emodins, napththazarins, and sterigmatocystins. However, it is worth highlighting that several categories without alerts showed a high predicted mutagenicity index, such as alternarenes, chromanes, enniatins and beauvericins, fusidic acids, nitropropionic acids, and verticillins. 

## 3. Discussion

In this work, we explored the application of in silico QSAR models to obtain toxicity data of mycotoxin, which are urgently needed for public health regulations. In order to obtain a general overview of the type and number of mycotoxins that exist, we performed a comprehensive search, generating for the first time a database containing almost 4400 compounds. Our research revealed that the number and diversity of the identified mycotoxins is much higher than generally assumed: most publications only indicate the existence of several hundreds of mycotoxins, while we found more than 4000 known mycotoxins. It is worth mentioning the high structural diversity of these compounds, which generally have relatively high molecular weights and many structures including sugar, aromatic, or peptide rings. 

The constructed database allowed us to perform a search of mutagenicity (Ames test), genotoxicity (in vivo and in vitro MN assay), and carcinogenicity (in vivo models) data by overlapping with different experimental databases. The search confirmed a low availability of experimental data, covering only a small percentage of the total existing mycotoxins. Nevertheless, we could obtain and validate robust QSAR models that predicted the four endpoints on an external validation set of mycotoxins with a relatively high accuracy, sensitivity, and specificity. These good results could be achieved by taking into account the specific characteristics of mycotoxins during the model building process, creating an appropriate applicability domain by the inclusion of mycotoxin and mycotoxin-like structure in the training set, and using a broad panel of 2D and 3D chemical descriptors. This was further confirmed by a comparison of the prediction of the mycotoxin’s validation set with three other, more general, QSAR reference tools (Table A11), which provided a worse prediction and only included part of the molecules in their applicability domain. In the case of the genotoxicity, the specificity of the predictions was moderate to low, probably due the fact that only a few data from non-genotoxic mycotoxins were retrieved and incorporated in the models. Thus, further experiments are required to confirm the existence of non-genotoxic categories of mycotoxins. However, following the caution principle, a model with lower specificity (predicting false positives) is preferred over a model with lower sensitivity (predicting false negatives).

When applying the prediction models to the database constructed containing almost 4400 mycotoxins, we obtained a very high proportion of mutagenic, carcinogenic, and especially genotoxic compounds. This result is not unexpected, as mycotoxins are defined *per se* as toxic compounds, and compounds of many categories proved to induce acute toxicity. However, differences between categories can be observed, mainly due to differences in the chemical structure. Concerning genotoxicity, all major categories included compounds with a positive genotoxicity prediction. Among them, some categories are well known to be genotoxic, such as aflatoxins, ochratoxins, or sterigmatocystins. Indeed, 97% of mycotoxins from the ochratoxin family have been predicted as genotoxic compounds. This result agrees with the scientific opinion published by the EFSA in 2020 [32] indicating the genotoxicity of ochratoxin A and thus eliminating the previously established TDI and establishing instead an MOE (Margin Of Exposure), as no threshold can be allowed for genotoxic compounds. In the case of sterigmatocystin, a mycotoxin structurally related to aflatoxin B1, it has been demonstrated to induce tumors in diverse animal species, and thus, it is a known carcinogen mycotoxin [33], which agrees with the prediction performed with our QSAR models. 

However, some categories showing a high percentage of genotoxic potential, are not well studied. For instance, griseofulvins were predicted as genotoxic by both in vitro and in vivo QSAR models. In the literature, animal studies have shown evidence that they are able to cause a variety of acute and chronic toxic effects, including liver and thyroid cancer in rodents, abnormal germ cell maturation, teratogenicity, and embryotoxicity in various species [34].

**Figure 6:**
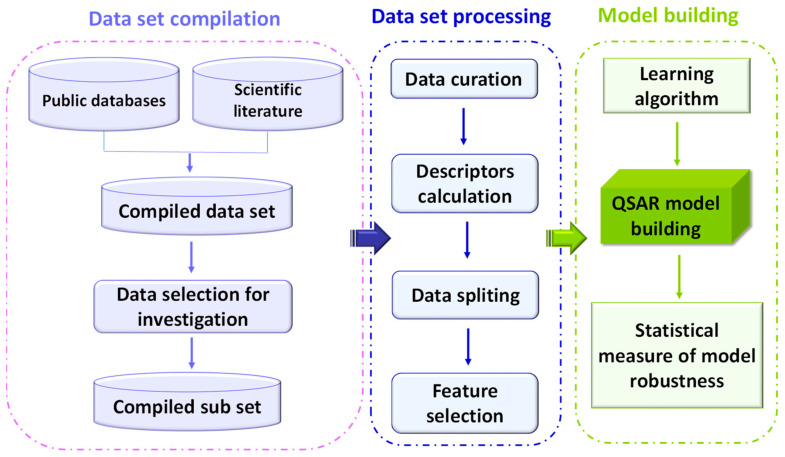
Workflow diagram for QSAR model building.

Regarding enniatins and beauvericins, commonly named as emerging *Fusarium* mycotoxins, the EFSA concluded in 2014 that a risk assessment was not possible given the lack of relevant toxicity data [35]. On one hand, in vitro genotoxicity data available suggested a potential genotoxic effect for beauvericin, while in vitro genotoxicity data for enniatins were negative. These results agree with those predicted by the in vitro QSAR model developed in our study (Figure 6). On the other hand, there are no in vivo genotoxicity data for either beauvericin or enniatins and no studies on carcinogenicity of beauvericin and enniatins have been identified, and thus, the use of in silico predictions for these endpoints can provide valuable information. Thus, according to predictions on enniatins and beauvericin, 79% and 71% of compounds from this category were predicted as genotoxic (in vivo model) and carcinogenic, respectively. In addition, 71% of enniatins and beauvericin were also predicted as mutagenic, thus suggesting a careful assessment of the emerging *Fusarium* mycotoxins toxicity.

Compounds from several categories have been classified as carcinogenic by the IARC [36], such as aflatoxins, trichothecenes, or fumonisins. Other classes, such as actinomycins, cyclosporings, and lovastatins, have still not been classified, but they are labelled as potentially carcinogenic by the ECHA. Furthermore, in several categories, carcinogenicity predictions proved to have an impact on the DNA of cells. For instance, aphidicolin is an inhibitor of eucaryotic nuclear DNA. Brevianamide produced a slightly teratogenic effect in chick embryos [37]. Emodin is suspected to create DNA strand breaks and/or non-covalently binding to DNA and inhibiting the catalytic activity of topoisomerase II (Toxin and Toxin Target Database (T3DB)); nevertheless, a genotoxic effect could be confirmed [38]. Mycophenolic acid inhibits the de novo pathway of guanosine nucleotide synthesis without incorporation into DNA (Toxin and Toxin Target Database (T3DB)). 

Alternariols and alternarenes are *Alternaria* mycotoxins that can be found in cereals around the world, but little relevance is still given to this fact. Currently, the toxicity of several altenariols is being investigated, including alternariol, alternariol monomethyl ether, altertoxins, altenuene, tenuazonic acid, and tentoxin. Among them, tenuazonic acid, alternariol, alternariol monomethyl ether, altenuene, and altertoxin I are the most important mycotoxins that can be found as contaminants in fruits and vegetables [39]. In 2011, the EFSA carried out a risk assessment on *Alternaria* toxins, as they were reported to induce genotoxicity, cytotoxicity, and reproductive and developmental toxicity, among other adverse effects [40]. Regarding their genotoxic effects, it was reported that alternariol, alternariol monomethyl ether, and altertoxins could induce gene locus mutation, DNA damage or synthesis disorder, chromosome aberration, and other effects in in vitro studies. In fact, according to the in vitro genotoxicity model developed, 100% of mycotoxins from the alternariols category was predicted as genotoxic compounds. In addition, alternariols have been related to the high incidence of esophageal cancer in Linxian, China [39], which can be related to our findings, as 62% of compounds belonging to this family were predicted as carcinogenic mycotoxins. Thus, special attention should be paid to this mycotoxin category. In this sense, maximum levels have been recently recommended by the EU in the Commission Recommendation 2022/553 [41] for alternariol, alternariol monomethyl ether, and tenuazonic acid. 

A high percentage of trichothecenes have been predicted as genotoxic by the in vitro model (97%) and the in vivo model (76%). The main trichothecene reported to occur in food commodities is deoxynivalenol. Although deoxynivalenol is not genotoxic by itself, it has recently been shown that this toxin exacerbates the genotoxicity induced by model or bacterial genotoxins. In addition, other trichothecenes, namely, T-2 toxin, diacetoxyscirpenol, nivalenol, fusarenon-X, and the newly discovered NX toxin, were also reported as compounds able to exacerbate the DNA damage inflicted by various genotoxins [42]. In addition, in the study reported by Yang et al. [43], deoxynivalenol was able to cause damage to the membrane, the chromosomes, and the DNA at all times of culture in human peripheral blood lymphocytes, thus concluding that deoxynivalenol potentially triggers genotoxicity in human lymphocytes. In other study performed on Sprague Dawley rats, deoxynivalenol increased the percentage of chromosomal aberration, DNA fragmentation, and comet score [44].

Citrinin, a mycotoxin classified in the chromanes category, has been reported to be genotoxic at high concentrations in cultured human lymphocytes, as it caused a significant concentration-dependent increase in MN frequency in human lymphocytes [45], a result according to our genotoxicity predictions for the chromanes category, where almost 90% of compounds were predicted as genotoxic by both the in vitro and the in vivo models.

The same occurs with zearalenones, which have been predicted as genotoxic by both genotoxicity models, according to some data reported showing that zearalenone and some of its metabolites increased the percentage of chromosome aberrations in mouse bone-marrow cells and in HeLa cells [46] and can increase the frequencies of polychromatic erythrocytes micronucleated and chromosomal aberrations in bone marrow cells from Balb/c female mice [47].

Regarding fumonisins, 87% of mycotoxins belonging to this family were predicted as genotoxic by the in vitro model. The most predominant, fumonisins, fumonisin B1, fumonisin B2, and fumonisin B3, are carcinogenic and genotoxic secondary metabolites found in corn-based foods worldwide and are produced by *Fusarium verticillioides* and *F. proliferatum* [48]. Fumonisin B1 is defined by IARC as a possible human carcinogen in Group 2B, and it shows genotoxic activity via oxidative stress, DNA damage, cell cycle arrest, apoptosis, inhibition of mitochondrial respiration, and deregulation of calcium homeostasis [49]. Some studies revealed that exposure to fumonisin B1 caused a significant increase in micronucleus frequency in a concentration- and time-dependent manner in rabbit kidney cells [50], and in HepG2 cells, fumonisin B1 has shown clastogenic effects [51].

Studies on the genotoxic activity of ergot alkaloids, also predicted as genotoxic by our models, are very limited. In the scientific report delivered by EFSA in 2012 [52], it was stated that genotoxicity studies on ergot alkaloids were insufficient, and more concretely, some studies evaluating the genotoxic and mutagenic effects of ergotamine revealed different results. In the literature, it has been reported that ergotamine is able to induce chromosomal abnormalities in human lymphocytes and leukocytes [53] but does not show mutagenic effects in mouse lymphoma cells [54]. Other authors have demonstrated that ergotamine and ergometry can induce sister chromatid exchange in ovarian cells [55]. Due to the scarce and different data obtained, further studies are necessary to evaluate the genotoxic and mutagenic potential of ergot alkaloids.

Furthermore, our results reveal that several mycotoxin categories are predicted as mutagenic but have no mutagenicity alert following ICH-M7 criteria. This did not happen when we performed the same comparison with a general database of organic compounds, where almost all molecules predicted as mutagenic had an alert. For some of these categories, no mutagenicity has been detected previously (alternarenes, enniatins and beauvericins, fusidic acids, and verticillins), while for others, some experimental evidence exists already (chromanes, nitropropionic acids, among others) [56,57]. This suggests that the structure of the mycotoxins could have been underestimated in the expert analysis of the mutagenicity, and that ICH-M7 criteria do not take into account specific mutagenic structural motives present in mycotoxins. Regulatory agencies should take this into account and request a revised version of these criteria to obtain a better coverage of mycotoxins, which are a danger for public health, and thus prioritize mycotoxins based on their mutagenic, genotoxic, and carcinogenic potential, as already suggested in other studies [58,59,60].

## 4. Conclusions

The web server developed in this work represents a valuable resource for scientists, industry, and regulatory agencies, including a comprehensive database of over 4000 mycotoxins divided into categories that can be easily explored by an interactive visualization. To our knowledge, this is the first database containing such a high number of mycotoxins. Furthermore, the user can directly access a prediction of the mutagenicity, genotoxicity, and carcinogenicity of the whole ensemble of mycotoxins, without the need to perform a prediction workflow. The data are based on mycotoxin specific and robust QSAR models, that were built up according to OECD principles and are adapted to REACH criteria, which means that they can be used for regulatory purposes. Thus, the developed models are a valuable tool for screening toxicity of non-regulated mycotoxins. Future perspectives of our work include the experimental validation of our models by analyzing selected mycotoxins from categories with no data in the training set, which have to be synthesized or purified, as most of them are not commercially available. Furthermore, the new QSAR models will be included in our ProtoPRED platform. 

## 5. Materials and Methods

To achieve the development of adequate and robust QSAR models, several elements are required. First of all, a data set providing experimental values of a biological activity or property for a group of already tested chemicals is necessary; in this case, the biological properties evaluated were mutagenicity, genotoxicity, and carcinogenicity; thus, experimental results derived from the mutagenic assay *Ames test* and the in vitro and in vivo MN test, and in vivo long-term carcinogenicity assay on rodents have been collected, as described in Section 5.1. Secondly, statistical methods (often called chemometric methods) are employed to find and validate the relationship between the calculated descriptors and the toxicity properties of the mycotoxins. The exact workflow is summarized in Figure 6 and described in the following sections.

### 5.1. Mycotoxin Data Set Construction and Chemical Data Curation 

For the construction of a general data set of mycotoxins, an initial list of mycotoxins was collected from the literature [22,61,62,63,64,65,66] and specific mycotoxin databases (https://zenodo.org/record/2648816#.ZClfI3ZBy5d; https://sciex.com/products/spectral-library/mycotoxin-libraries) (accessed on 2 March 2022).

This list was further completed by searching in PubChem for compounds of the same family, and their metabolites, resulting in a list of 4360 compounds. Only compounds that were directly produced by fungi and their metabolites were included. For each compound, the isomeric SMILES together with the CAS number and the PubChem CID code was retrieved. The resulting database was further curated by normalizing the smiles and removing counterions, salts, and mixtures. Finally, each compound was assigned to a specific family or category to their chemical structure, obtaining 170 different categories (Table A1). 

### 5.2. Mycotoxin Clustering and Chemical Space Distribution 

To study the chemical space of the generated mycotoxins data set, a fingerprinting system was generated using the LSH Forest algorithm from TMAP (https://tmap.gdb.tools/, accessed on 20 May 2023) for dimensionality reduction. Each point in the TMAP represents a fingerprint of a unique chemical transformation generated using the fully trained model. The points were colored by categories or their outcome in the toxicity predictions. The Faerun package (https://pypi.org/project/faerun/, accessed on 20 May 2023) was used to create an interactive visualization tool for the clustering scheme.

### 5.3. Mycotoxin Mutagenicity QSAR Model

**Figure 7:**
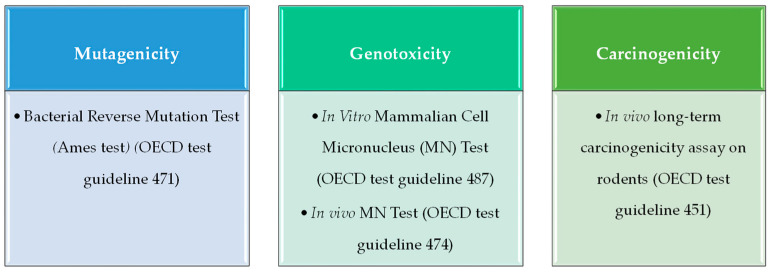
In vitro and in vivo assays consulted for model development and their corresponding OECD test guideline.

For QSAR mutagenicity model development, the endpoint Bacterial Reverse Mutation Test (Ames test) (OECD test guideline 471) was selected (Figure 7), considered the first outcome to assess the possible mutagenicity of a substance [67,68]. We scanned several high-quality databases (Carcinogenic Potency Database CEBS, CCRIS Mutagenicity assay, Vega, QSAR Toolbox, EFSA OpenFoodTox, ECVAM, etc.) as well as the scientific literature [68] for data of the compounds of our previously generated mycotoxin database. For each compound, CAS number, isomeric SMILES, and experimental data expressed as active (1) or inactive (0) were compiled in a table.

As a result, raw data of 356 mycotoxins were recovered, which were further refined as follows: For a first model A, data of 120 compounds (75 mutagens and 45 no mutagens) were selected that strictly fulfil the OECD guideline TG471: assayed with at least five *Salmonella* strains (TA1535, TA1537 or TA97 or TA 97a, TA98, TA100, and TA102) with and without microsomal activation by S9 fraction, since often the interaction with genetic material occurs after metabolic activation. For a second model B, data were selected less strictly, to cover a greater part of the chemical space, including data from at least two strains with or without metabolic activation. The data set of model B was composed of 287 compounds, with a ratio of mutagens to non-mutagens of 108/179. A third data set of 24 compounds, fulfilling the OECD guideline, was saved for external validation (Section 5.6).

For the development of the QSAR models, around 4000 different chemical descriptors from 15 different categories were calculated for each compound with an in-house python script. Then, descriptors were selected by recursive feature elimination (RFE) to 7 for model A and 13 for model B (Table A2 and Table A3). In the next step, the data set was divided into a training set for model building, and a test set, in a proportion of 75–25%, respectively. Mutagenic and non-mutagenic compounds were distributed homogeneously in both sets. For model building, several algorithms were tested on the training set, obtaining the best metrics for Logistic Regression. 

### 5.4. Mycotoxin Genotoxicity QSAR Models

For in vitro genotoxicity QSAR modelling, the in vitro MN assay was chosen, a robust and quantitative assay of chromosome damage with the capacity to detect not only clastogenic and aneugenic events but also some epigenetic effects and recommended by the OECD for genotoxicity evaluation in test guideline 487 (Figure 7). 

Databases (Genetic Toxicology Data Bank in PubChem and eChemPortal) and the scientific literature [69,70,71] were scanned for data for the in vitro MN assay, fulfilling the OECD guideline, retrieving data for 91 compounds, of which 15 were saved for external validation (Section 5.6). To enable model building, the data set was further completed with 379 compounds that were not mycotoxins but close in the chemical space, generating a final data set of 455 compounds. The ratio of genotoxic to non-genotoxic compounds was 264/191, respectively. For model building, the same procedure as in Section 5.3 was employed, also obtaining model based on 25 descriptors (Table A4) based on Logistic Regression. 

For in vivo genotoxicity QSAR modelling, the in vivo MN assay on mammalian erythrocyte was chosen (OECD test guideline 474), which identifies substances that cause micronuclei in erythroblasts sampled from bone marrow and/or peripheral blood cells of animals, usually rodents. As not enough data for mycotoxins could be retrieved (72 mycotoxins from the ISSMIC public database), the QSAR model from ProtoPRED (https://protopred.protoqsar.com/, accessed on 20 May 2023) including mycotoxins in the training set, was applied. The model was built with 13 descriptors (Table A5) and based on the Support Vector Machine Classifier (SVC) algorithm. The model was then externally validated with the external set of 72 mycotoxins obtaining good results. All mycotoxins were included in the applicability domain of the model. 

### 5.5. Mycotoxin Carcinogenicity QSAR Model

For carcinogenicity assessment, the long-term carcinogenicity study on rat was employed, fulfilling the OECD test guideline 451 (Figure 7). As there was not enough data to build a specific model for mycotoxins (75 mycotoxins from different databases, such as PubChem AID_1259411, Carcinogenic Potency Database CEBS and OpenFoodTox TX22525), the QSAR model from ProtoPRED (https://protopred.protoqsar.com/ accessed on 20 May 2023) was applied, showing excellent results when applied to the 75 mycotoxins in the external validation. All mycotoxins were included in the applicability domain of the model. 

The data for developing the model was extracted from Carcinogenicity I (ISSCAN) public database retrieved from QSAR Toolbox, which contains curated information on chemical compounds tested with the long-term carcinogenicity bioassay on rodents (rat and mouse). The main primary sources of data are the NTP, CPDB, CCRIS, and IARC repositories. After curation and preprocessing, the database was formed by 652 experimental results, with 251 of positive values (38.5%) and 401 negative values (61.5%). For model building, the same procedure as in Section 5.3 was employed, also obtaining the best metrics for Light Gradient Boosting Machine (LGBM) Classifier.

### 5.6. External Model Validation

The predictive potential of the QSAR models was evaluated with a completely independent data set of mycotoxins, in terms of accuracy, precision, sensitivity, and specificity. The metrics considered to evaluate the performance of the built classification models were calculated according to the following formulas:

Accuracy = TP + TN/(TP + FN + FP + TN)

Precision = TP/(TP + FP)

Sensitivity = TP/(TP + FN)

Specificity = TN/(TN + FP)

where TP = true positive; FP = false positive; TN = true negative; and FN = false negative.

Details about the compounds selected for each validation can be found in Table A7, Table A8, Table A9 and Table A10. 

## Data Availability

The mycotoxin database and all performed predictions can be downloaded free of charge at the webserver: https://chemopredictionsuite.com/MicotoXilico.

## Appendix A

Table A1, Table A2, Table A3, Table A4, Table A5, Table A6, Table A7, Table A8, Table A9, Table A10 and Table A11.

**Table A1:** Number of compounds in each mycotoxin category.

Category	Num of Cpds
trichothecenes	410
ergot alkaloids	158
cytochalasan alkaloids	147
actinomycins	141
resveratrols	121
aphidicolins	90
nucleotides	88
not classified	79
alkaloids	74
emodins	67
taxols	67
fumonisins	63
zearalenones	60
peptides	59
cyclosporins	58
lovastatins	58
aflatoxins	57
coumarins	55
nitropropionic acids	54
ophiobolins	54
terpenoids	51
penicillins	50
triterpenoids	46
destruxins	41
brefeldins	38
phenols	37
fumitremorgins	36
brevianamides	36
naphthazarins	36
alternarenes	36
ETPs	34
anthraquinones	34
brassicicenes	34
ochratoxins	33
sterigmatocystins	33
mycophenolic acids	32
penitrems	32
griseofulvins	32
pyropenes	32
verticillins	31
fusidic acids	31
fatty acid like	29
cyclic peptides	29
decarestrictines	28
chromanes	27
naphthalenes	25
yanuthones	25
benzene derivatives	25
furans	25
alternariolides	24
viridins	24
alternariols	24
quinazolines	24
roquefortines	23
anthracyclines	22
territrems	22
radicicols	22
asterriquinones	22
altersolanols	21
pyrones	21
xanthones	21
apicidins	20
enniatins	19
chinulins	19
rugulosins	19
tyrosols	19
beauvericins	19
wortmannins	19
austalides	19
pyrenocines	19
cyclopeptins	18
chaetomugilins	18
zaragozic acids	18
chrysophanols	18
alterporriols	18
rubrofusarins	17
gibberellins	17
andrastins	16
physcions	16
aspernolides	16
abscisic acids	15
koninginins	15
roridins	14
versicolorins	14
chetomins	14
malformins	14
radicinins	14
usnic acids&ustins	13
secalonic acids	13
aspewentins	13
gregatins	13
altertoxins	13
aspulvinones	13
phomopsins	12
rubellins	12
viridicatins	12
cotylenins	12
aureobasidins	12
communesins	12
altenuenes	12
austocystins	12
austins	12
arugosins	12
fusaric acids	11
patulins	11
lactones	11
fusarins&fusarielins	11
zinniols	11
cladosporols	11
altenusins	11
emestrins	11
schweinfurthins	10
fumagillins	10
aurasperones	10
astellolides	10
penicillenols	10
tryptophols	10
kojic acids	10
averufins	10
culmorins	10
kipukasins	10
xalines	10
rubratoxins	9
brevicompanines	9
penostatins	9
ustiloxins	9
melleins	9
sphingofungins	8
asperlins&asperlactones	8
thiazolidines	8
benzoquinones	8
nigerapyrones	8
andibenins	8
cordycepins	8
asterriquinones	8
chartarlactams	8
vertinols	7
tenuazonic acids	7
xanthins	7
calphostins	7
erythromycins	7
marcfortines	7
bikaverins	7
solanapyrones	7
quinolones	7
cephalosporins	7
stachybocins	7
chevalones	7
glaucins	7
fusarielins	6
phtalates	6
depudecins	6
meleagrins	6
myrothecines	6
AF toxins	6
aflavinines	6
cercosporins	5
chromanones	5
aspergillimides	5
tryprostatins	5
amino acid derivative	5
flavones	4
chlamydosporols	4
amphotericins	4
aspilactonols	4
emodins&rubrofusarins	4
orienticins	4
candidusins	4
asperpyrones	3
candidins	3

**Table A2:** Molecular descriptors selected by recursive feature elimination (RFE) for QSAR mutagenicity model A.

Descriptor	Definition
C-030	X–CH–X atom-centered fragments
ATSC7Z	centered Moreau–Broto autocorrelation of lag 7 weighted by atomic number
ATSC8dv	centered Moreau–Broto autocorrelation of lag 8 weighted by valence electrons
MATS1c	Moran coefficient of lag 1 weighted by Gasteiger charge
GATS1c	Geary coefficient of lag 1 weighted by Gasteiger charge
GATS3d	Geary coefficient of lag 3 weighted by sigma electrons
X1solA	average solvation connectivity index of order 1

**Table A3:** Molecular descriptors selected by recursive feature elimination (RFE) for QSAR mutagenicity model B.

Descriptor	Definition
C-018	=CHX atom-centered fragments
C-019	=CRX atom-centered fragments
ATSC6dv	centered Moreau–Broto autocorrelation of lag 6 weighted by valence electrons
ATSC8dv	centered Moreau–Broto autocorrelation of lag 8 weighted by valence electrons
AATSC5p	averaged and centered Moreau–Broto autocorrelation of lag 5 weighted by polarizability
MATS1c	Moran coefficient of lag 1 weighted by Gasteiger charge
nR_3_False_False_False_True	number of 3–membered rings non-aromatic hetero
R1s+	R maximal autocorrelation of lag 1/weighted by I-state GETAWAY descriptors
NPR2	normalized principal moment of order 2
De	D total accessibility index, Sanderson electronegativity-weighted
Mor28v	signal 28/weighted by van der Waals volume 3D-MoRSE descriptors
Mor02s	signal 02/weighted by I-state 3D-MoRSE descriptors
Mor23s	signal 23/weighted by I-state 3D-MoRSE descriptors

**Table A4:** Molecular descriptors selected by recursive feature elimination (RFE) for QSAR in vitro genotoxicity model.

Descriptor	Definition
H-047	atom-centered fragments. H attached to C^1^(sp^3^)/C^0^(sp^2^)
O-056	alcohol. Atom-centered fragments
O-061	O–atom-centered fragments
F-084	F attached to C1(sp2). Atom-centered fragments
AATS5dv	averaged Moreau–Broto autocorrelation of lag 5 weighted by valence electrons. 2D
ATSC4v	centered Moreau–Broto autocorrelation of lag 4 weighted by van der Waals volume 2D autocorrelations
AATSC1Z	averaged and centered Moreau–Broto autocorrelation of lag 1 weighted by atomic number. 2D
AATSC5c	averaged and centered Moreau–Broto autocorrelation of lag 5 weighted by Gasteiger charge. 2D
GATS2Z	Geary coefficient of lag 2 weighted by atomic number. 2D
BELdv0	highest eigenvalue of Burden matrix weighted by valence electrons
L3i	3rd component size directional WHIM index/weighted by ionization potential WHIM descriptors
Mor09u	signal 09/unweighted 3D-MoRSE descriptors
Mor22s	signal 22/weighted by I-state 3D-MoRSE descriptors
JhetZ	Balaban-type index from *Z*-weighted distance matrix (Barysz matrix)
D/Dr3	distance/detour ring index of order 3
JGI9	mean topological charge index of order 9 2D autocorrelations
B05[O-O]	presence/absence of O-O at topological distance 5 2D Atom Pairs
B07[C-N]	presence/absence of C-N at topological distance 7 2D Atom Pairs
B08[C-O]	presence/absence of C-O at topological distance 8 2D Atom Pairs
B10[C-C]	presence/absence of C-C at topological distance 10 2D Atom Pairs
nAllOx	number of allylic oxidation sites excluding steroid dienone
nByciclic	number of atoms that are in two rings.
PEOE_VSA4	MOE Charge VSA Descriptor 4 (−0.20 <= x < −0.15). 2D
SLogP_VSA4	MOE logP VSA Descriptor 4 (0.00 <= x < 0.10). 2D

**Table A5:** Molecular descriptors selected by Recursive Feature Elimination (RFE) based on Support Vector Machine (SVM) using linear kernel (C = 1), for QSAR in vivo genotoxicity model.

Descriptor	Definition
C-016	”=CHR.”
ATSC5i	centered Moreau–Broto autocorrelation of lag 5 (log function) weighted by ionization potential.
AATSC0se	averaged centered Moreau–Broto autocorrelation of lag 0 (log function) weighted by Sanderson electronegativity.
AATSC5i	averaged centered Moreau–Broto autocorrelation of lag 5 (log function) weighted by ionization potential.
MATS4p	Moran autocorrelation of lag 4 (log function) weighted by polarizability.
GATS4i	Geary autocorrelation of lag 4 (log function) weighted by ionization potential.
B02(C-C)	presence/absence of C-C at topological distance 2.
B02(C-O)	presence/absence of C-O at topological distance 2.
B04(C-O)	presence/absence of C-O at topological distance 4.
B04(O-O)	presence/absence of O-O at topological distance 4.
B08(C-N)	presence/absence of C-N at topological distance 8.
B09(O-O)	presence/absence of O-O at topological distance 9.
EState_VSA6	EState VSA descriptor 6.

**Table A6:** Molecular descriptors selected by Light Gradient Boosting Machine (LGBM) for QSAR carcinogenicity model.

Descriptor	Definition
AATSC1dv	averaged centered Moreau–Broto autocorrelation of lag 1 (log function) weighted by valence electrons
SMR_VSA9	MOE MR VSA descriptor 9
SIC2	structural information content index (neighborhood symmetry of 2-order)
ATSC1pe	centered Moreau–Broto autocorrelation of lag 1 (log function) weighted by pauling EN
C-032	X–CX–X
JGI7	mean topological charge index of order 7
BELc0	highest eigenvalue of Burden matrix weighted by charge
AATSC4se	averaged centred Moreau–Broto autocorrelation of lag 4 (log function) weighted by Sanderson electronegativity
GATS6se	Geary autocorrelation of lag 6 (log function) weighted by Sanderson electronegativity
NssNH	number of ssNH
D/Dr5	distance/detour ring index of order 5
GATS1s	Geary autocorrelation of lag 1 (log function) weighted by I-state
RBF	rotatable bond fraction
JGI5	mean topological charge index of order 5
PJI2	2D Petitjean shape index
GATS1d	Geary autocorrelation of lag 1 (log function) weighted by sigma electrons
AATSC3d	averaged centred Moreau–Broto autocorrelation of lag 3 (log function) weighted by sigma electrons

**Table A7:** Validation set for mutagenicity data.

SMILES	Name	y
CC1=C[C@H]2[C@@H](CC3=CNC4=CC=CC2=C34)N(C1)C	agroclavine	0
C[C@H]1CCCC(=O)CCC/C=C/C2=C(C(=CC(=C2)O)O)C(=O)O1	zearalenone	0
CC1=C[C@@H]2[C@](CC1)([C@]3([C@@H]([C@H]([C@H]([C@@]34CO4)O2)O)OC(=O)C)C)COC(=O)C	diacetoxyscirpenol	0
CC1=C[C@@H]2[C@]([C@@H](C1=O)O)([C@]3(C[C@H]([C@H]([C@@]34CO4)O2)O)C)CO	deoxynivalenol	0
C[C@@H]1[C@H](O1)[C@H]2[C@H](C=CC(=O)O2)OC(=O)C	asperlin	0
CC(C)[C@@]1(C(=O)N2[C@H](C(=O)N3CCC[C@H]3[C@@]2(O1)O)CC4=CC=CC=C4)NC(=O)[C@@H]5C[C@H]6[C@@H](CC7=CNC8=CC=CC6=C78)N(C5)C	dihydroergocristine	0
CCCC[C@@H](C)[C@H]([C@H](C[C@@H](C)C[C@@H](CCCC[C@H](C[C@@H]([C@H](C)N)O)O)O)OC(=O)C[C@@H](CC(=O)O)C(=O)O)OC(=O)C[C@@H](CC(=O)O)C(=O)O	fumonisin B1	0
CCCC[C@@H](C)[C@H]([C@H](C[C@@H](C)CCCCCC[C@H](C[C@@H]([C@H](C)N)O)O)OC(=O)C[C@@H](CC(=O)O)C(=O)O)OC(=O)C[C@@H](CC(=O)O)C(=O)O	fumonisin B2	0
CCCC[C@@H](C)[C@H]([C@H](C[C@@H](C)C[C@@H](CCCCCC[C@H]([C@H](C)N)O)O)OC(=O)CC(CC(=O)O)C(=O)O)OC(=O)CC(CC(=O)O)C(=O)O	fumonisn B3	0
CC1=C[C@@H]2[C@]([C@@H](C1=O)O)([C@]3([C@@H]([C@H]([C@H]([C@@]34CO4)O2)O)OC(=O)C)C)CO	fusarenon-X	0
CC1=C[C@@H]2[C@](CC1)(C3([C@@H]([C@H]([C@H](C34CO4)O2)O)OC(=O)C)C)CO	4-Acetoxyscirpenol	0
CC1=C2COC(=O)C2=C(C(=C1OC)C/C=C(\C)/CCC(=O)O)O	mycophenolic acid	0
COC1=C2C3=C(C(=O)CC3)C(=O)OC2=C4[C@@H]5CCO[C@@H]5OC4=C1	aflatoxin B2	1
C[C@H]1[C@H]([C@H](C[C@@H](O1)O[C@H]2C[C@@](CC3=C2C(=C4C(=C3O)C(=O)C5=CC=CC=C5C4=O)O)(C(=O)C)O)N)O	idarubicin	1
C[C@@H]1[C@@H](C(=O)N[C@@H](C(=O)N2CCC[C@H]2C(=O)N(CC(=O)N([C@H](C(=O)O1)C(C)C)C)C)C(C)C)NC(=O)C3=C4C(=C(C=C3)C)OC5=C(C(=O)C(=C(C5=N4)C(=O)N[C@H]6[C@H](OC(=O)[C@@H](N(C(=O)CN(C(=O)[C@@H]7CCCN7C(=O)[C@H](NC6=O)C(C)C)C)C)C(C)C)C)N)C	actinomycin D	1
COC1=C2C3=C(C(=O)CC3)C(=O)OC2=C4[C@@H]5[C@@H]6[C@@H](O6)O[C@@H]5OC4=C1	2,3-Epoxyaflatoxin B1	1
COC1=C2C3=C(C(=O)CC3)C(=O)OC2=C4C5CCOC5OC4=C1	aflatoxin B2 alpha	1
COC1=C2C3=C([C@@H](CC3)O)C(=O)OC2=C4[C@@H]5C=CO[C@@H]5OC4=C1	aflatoxicol B	1
COC1=C2C(=C3[C@@H]4C=CO[C@@H]4OC3=C1)OC5=CC=CC(=C5C2=O)O	sterigmatocystin	1
COC1=C2C3=C(C(=O)C[C@@H]3O)C(=O)OC2=C4[C@@H]5C=CO[C@@H]5OC4=C1	aflatoxin Q1	1
C[C@H]1[C@H]([C@H](C[C@@H](O1)O[C@H]2C[C@@](CC3=C2C(=C4C(=C3O)C(=O)C5=C(C4=O)C(=CC=C5)OC)O)(/C(=N\NC(=O)C6=CC=CC=C6)/C)O)N)O	zorubicin	1
C1=NC(=C2C(=N1)N(C=N2)[C@H]3[C@@H]([C@@H]([C@H](O3)CO)O)O)NO	inosine oxime	1
C[C@@H]1[C@@]2([C@@H](O2)[C@](O1)(C)/C=C(\C)/C=C(\C)/[C@H]3[C@](O3)(C)C4=C(C(=C(C(=O)O4)C)OC)C)C	verrucosidin	1
C1C[C@]2([C@@H]3[C@H](CC(=O)C4=C(C=CC(=C34)C5=C2C(=C(C=C5)O)C1=O)O)O)O	altertoxin I	1

**Table A8:** Validation set for in vitro genotoxicity data.

SMILES	Name	y
CC(C)C[C@H]1C(=O)O[C@@H](C(=O)N([C@H](C(=O)O[C@@H](C(=O)N([C@H](C(=O)O[C@@H](C(=O)N1C)C(C)C)CC(C)C)C)C(C)C)CC(C)C)C)C(C)C	enniatin C	0
CC[C@H](C)[C@@H]1C(=O)N([C@H](C(=O)O[C@@H](C(=O)N([C@H](C(=O)O[C@@H](C(=O)N([C@H](C(=O)O1)C(C)C)C)[C@@H](C)CC)C(C)C)C)C(C)C)C(C)C)C	enniatin I	0
CC[C@H](C)[C@H]1C(=O)O[C@@H](C(=O)N([C@H](C(=O)O[C@@H](C(=O)N([C@H](C(=O)O[C@@H](C(=O)N1C)C(C)C)C)C)C(C)C)C(C)C)C)C(C)C	enniatin J3	0
CC[C@H](C)[C@@H]1C(=O)N([C@H](C(=O)O[C@@H](C(=O)N([C@H](C(=O)O[C@@H](C(=O)N([C@H](C(=O)O1)C(C)C)C)C(C)C)C(C)C)C)C(C)C)C(C)C)C	enniatin H	0
CC[C@H]1C(=O)O[C@@H](C(=O)N([C@H](C(=O)O[C@@H](C(=O)N([C@H](C(=O)O[C@@H](C(=O)N1C)C(C)C)C(C)C)C)C(C)C)C(C)C)C)C(C)C	enniatin K1	0
CCC(C)[C@@H]1C(=O)N([C@H](C(=O)O[C@@H](C(=O)N([C@H](C(=O)O[C@@H](C(=O)N([C@H](C(=O)O1)C(C)C)C)C(C)C)C(C)C)C)C(C)C)C(C)C)C	cyclo[D-OVal-N(Me)Val-D-OVal-N(Me)Val-D-OxiIle-N(Me)Val]	0
O=c1cc(CO)occ1O	kojic acid	0
CC1=C[C@@H]2[C@]([C@@H](C1=O)O)([C@]3(C[C@H]([C@H]([C@@]34CO4)O2)O)C)COC(=O)C	15-acetyldeoxynivalenol	1
COC1=CC2=C(C(=C1)OC)C(=O)C3=C(C4=C(C=C3C2=O)O[C@@H]5[C@H]4CCO5)O	6,8-O-Dimethylversicolorin	1
COC1=C2C3=C(C(=O)CC3)C(=O)OC2=C4[C@@H]5C=CO[C@@H]5OC4=C1	aflatoxin B1	1
C[C@@H]1CCC[C@H](/C=C/C(=O)O[C@]23[C@@H](/C=C/C1)[C@@H](C(=C)[C@H]([C@H]2[C@@H](NC3=O)CC4=CC=CC=C4)C)O)O	cytochalasin B	1
C1CO[C@H]2[C@@H]1C3=C(O2)C=C(C4=C3OC5=CC=CC(=C5C4=O)O)O	demethyldihydrosterigmatocystin	1
CCCC[C@@H](C)[C@H]([C@H](C[C@@H](C)C[C@@H](CCCC[C@H](C[C@@H]([C@H](C)N)O)O)O)OC(=O)C[C@@H](CC(=O)O)C(=O)O)OC(=O)C[C@@H](CC(=O)O)C(=O)O	fumonisin B1	1
C1=CO[C@H]2[C@@H]1C3=C(O2)C=C4C(=C3O)C(=O)C5=C(C4=O)C=C(C=C5O)O	versicolorin A	1
C[C@H]1CCC[C@@H](CCC/C=C/C2=C(C(=CC(=C2)O)O)C(=O)O1)O	alpha-zearalenol	1

**Table A9:** Validation set for in vivo genotoxicity data.

SMILES	Name	y
Cc1cc(O)cc2oc(=O)c3c(O)cc(O)cc3c12	alternariol	0
CCCCCCCCCCCCCCC(C(CO)N)O	C17-Sphinganine	0
C/C=C/C[C@@H](C)[C@@H](O)[C@H]1C(=O)N[C@@H](CC)C(=O)N(C)CC(=O)N(C)[C@@H](CC(C)C)C(=O)N[C@@H](C(C)C)C(=O)N(C)[C@@H](CC(C)C)C(=O)N[C@@H](C)C(=O)N[C@H](C)C(=O)N(C)[C@@H](CC(C)C)C(=O)N(C)[C@@H](CC(C)C)C(=O)N(C)[C@@H](C(C)C)C(=O)N1C	cyclosporin A	0
CC1=C[C@H]2O[C@@H]3[C@H](O)C[C@@](C)([C@]34CO4)[C@@]2(CO)[C@H](O)C1=O	deoxynivalenol	0
C=C1C[C@]23C[C@@]1(O)CC[C@H]2[C@@]12C=C[C@H](O)[C@@](C)(C(=O)O1)[C@H]2[C@@H]3C(=O)O	gibberellic acid	0
O=c1cc(CO)occ1O	kojic acid	0
CN1C[C@@H](C=C2[C@H]1CC3=CNC4=CC=CC2=C34)C(=O)O	lysergic acid	0
CCN(CC)C(=O)[C@H]1CN([C@@H]2CC3=CNC4=CC=CC(=C34)C2=C1)C	lysergic acid diethylamine	0
CC(=O)OC[C@]12C[C@H](OC(=O)CC(C)C)C(C)=C[C@H]1O[C@@H]1[C@H](O)[C@@H](OC(C)=O)[C@@]2(C)[C@@]12CO2	T-2 toxin	0
CC(=O)OC[C@@]12C[C@@H](OC(=O)CC(C)C)C(C)=C[C@@H]1O[C@H]1[C@H](O)[C@@H](O)[C@]2(C)[C@@]12CO2	HT-2 toxin	0
CC(C)C[C@@H]1NC(=O)[C@H](C)N(C)C(=O)CNC(=O)/C(=C/c2ccccc2)N(C)C1=O	tentoxin	0
CC[C@H](C)[C@@H]1NC(=O)C(C(C)=O)=C1O	tenuazonic acid	0
CCC(C)C1NC(=O)C(C(C)=O)=C1O	tenuazonic acid	0
O=c1[nH]c(=O)n([C@H]2C[C@H](O)[C@@H](CO)O2)cc1Br	5-bromo-2′-deoxyuridine	1
Cc1c2oc3c(C)ccc(C(=O)NC4C(=O)NC(C(C)C)C(=O)N5CCC[C@H]5C(=O)N(C)CC(=O)N(C)C(C(C)C)C(=O)OC4C)c3nc-2c(C(=O)NC2C(=O)NC(C(C)C)C(=O)N3CCCC3C(=O)N(C)CC(=O)N(C)C(C(C)C)C(=O)OC2C)c(N)c1=O	A-2600	1
Cc1c2oc3c(C)ccc(C(=O)NC4C(=O)N[C@H](C(C)C)C(=O)N5CCC[C@H]5C(=O)N(C)CC(=O)N(C)[C@@H](C(C)C)C(=O)O[C@@H]4C)c3nc-2c(C(=O)NC2C(=O)N[C@H](C(C)C)C(=O)N3CCC[C@H]3C(=O)N(C)CC(=O)N(C)[C@@H](C(C)C)C(=O)O[C@@H]2C)c(N)c1=O	actinomycin D	1
Cc1c2oc3c(C)ccc(C(=O)NC4C(=O)NC(C(C)C)C(=O)N5CCCC5C(=O)N(C)CC(=O)N(C)C(C(C)C)C(=O)OC4C)c3nc-2c(C(=O)NC2C(=O)NC(C(C)C)C(=O)N3CCCC3C(=O)N(C)CC(=O)N(C)C(C(C)C)C(=O)OC2C)c(N)c1=O	actinomycin D	1
Cc1c2oc3c(C)ccc(C(=O)N[C@@H]4C(=O)N[C@H](C(C)C)C(=O)N5CCC[C@H]5C(=O)N(C)CC(=O)N(C)[C@@H](C(C)C)C(=O)O[C@H]4C)c3nc-2c(C(=O)N[C@@H]2C(=O)N[C@H](C(C)C)C(=O)N3CCC[C@H]3C(=O)N(C)CC(=O)N(C)[C@@H](C(C)C)C(=O)O[C@H]2C)c(N)c1=O	actinomycin D	1
Cc1c2oc3c(C)ccc(C(=O)N[C@@H]4C(=O)N[C@H](C(C)C)C(=O)N5CCC[C@H]5C(=O)N(C)CC(=O)N(C)[C@H](C(C)C)C(=O)O[C@@H]4C)c3nc-2c(C(=O)N[C@@H]2C(=O)N[C@H](C(C)C)C(=O)N3CCC[C@H]3C(=O)N(C)CC(=O)N(C)[C@@H](C(C)C)C(=O)O[C@@H]2C)c(N)c1=O	actinomycin D	1
Cc1c2oc3c(C)ccc(C(=O)N[C@@H]4C(=O)N[C@@H](C(C)C)C(=O)N5CCC[C@H]5C(=O)N(C)CC(=O)N(C)[C@@H](C(C)C)C(=O)O[C@@H]4C)c3nc-2c(C(=O)N[C@@H]2C(=O)N[C@@H](C(C)C)C(=O)N3CCC[C@H]3C(=O)N(C)CC(=O)N(C)[C@@H](C(C)C)C(=O)O[C@@H]2C)c(N)c1=O	actinomycin D	1
Cc1c2oc3c(C)ccc(C(=O)N[C@@H]4C(=O)N[C@H](C(C)C)C(=O)N5CCC[C@H]5C(=O)N(C)CC(=O)N(C)[C@@H](C(C)C)C(=O)O[C@@H]4C)c3nc-2c(C(=O)N[C@H]2C(=O)N[C@H](C(C)C)C(=O)N3CCC[C@@H]3C(=O)N(C)CC(=O)N(C)[C@@H](C(C)C)C(=O)O[C@@H]2C)c(N)c1=O	actinomycin D	1
Cc1c2oc3c(C)ccc(C(=O)N[C@@H]4C(=O)N[C@H](C(C)C)C(=O)N5CCC[C@H]5C(=O)N(C)CC(=O)N(C)C(C(C)C)C(=O)O[C@@H]4C)c3nc-2c(C(=O)N[C@@H]2C(=O)N[C@H](C(C)C)C(=O)N3CCC[C@H]3C(=O)N(C)CC(=O)N(C)[C@@H](C(C)C)C(=O)O[C@@H]2C)c(N)c1=O	actinomycin D	1
[3H]c1c([3H])c(C([3H])([3H])[3H])c2oc3c(C([3H])([3H])[3H])c(=O)c(N([3H])[3H])c(C(=O)N([3H])[C@]4([3H])C(=O)N([3H])[C@]([3H])(C([3H])(C([3H])([3H])[3H])C([3H])([3H])[3H])C(=O)N5C([3H])([3H])C([3H])([3H])C([3H])([3H])[C@]5([3H])C(=O)N(C([3H])([3H])[3H])C([3H])([3H])C(=O)N(C([3H])([3H])[3H])[C@@]([3H])(C([3H])(C([3H])([3H])[3H])C([3H])([3H])[3H])C(=O)O[C@]4([3H])C([3H])([3H])[3H])c-3nc2c1C(=O)N([3H])[C@]1([3H])C(=O)N([3H])[C@]([3H])(C([3H])(C([3H])([3H])[3H])C([3H])([3H])[3H])C(=O)N2C([3H])([3H])C([3H])([3H])C([3H])([3H])[C@]2([3H])C(=O)N(C([3H])([3H])[3H])C([3H])([3H])C(=O)N(C([3H])([3H])[3H])[C@@]([3H])(C([3H])(C([3H])([3H])[3H])C([3H])([3H])[3H])C(=O)O[C@]1([3H])C([3H])([3H])[3H]	actinomycin d-[3h(g)]	1
COc1cc2c(c3oc(=O)c4c(c13)CCC4=O)[C@@H]1C=CO[C@@H]1O2	aflatoxin B1	1
COc1cc2c(c3oc(=O)c4c(c13)CCC4=O)[C@@H]1CCO[C@@H]1O2	aflatoxin B2	1
COc1cc2c(c3oc(=O)c4c(c13)CCOC4=O)[C@H]1C=CO[C@H]1O2	aflatoxin G1	1
COc1cc2c(c3oc(=O)c4c(c13)CCOC4=O)[C@H]1C=CO[C@@H]1O2	aflatoxin G1	1
COc1cc2c(c3oc(=O)c4c(c13)CCOC4=O)[C@@H]1C=CO[C@@H]1O2	aflatoxin G1	1
COc1cc2c(c3oc(=O)c4c(c13)CCOC4=O)C1C=COC1O2	aflatoxin G1	1
COc1cc2c(c3oc(=O)c4c(c13)CCOC4=O)[C@@H]1CCO[C@@H]1O2	aflatoxin G2	1
COc1cc2c(c3oc(=O)c4c(c13)CCC4=O)[C@]1(O)C=CO[C@@H]1O2	aflatoxin M1	1
COc1cc2c(c3oc(=O)c4c(c13)[C@@H](O)CC4=O)[C@@H]1C=CO[C@@H]1O2	aflatoxin Q1	1
COc1cc2c(c3oc(=O)c4c(c13)CCC4O)[C@@H]1C=CO[C@@H]1O2	aflatoxicol	1
COc1cc2c(c3oc(=O)c4c(c13)CC[C@H]4O)[C@@H]1C=CO[C@H]1O2	aflatoxicol	1
COc1cc2c(c3oc(=O)c4c(c13)CC[C@@H]4O)[C@@H]1C=CO[C@@H]1O2	aflatoxicol	1
C[C@@H]1CCC[C@H](O)CCCCCc2cc(O)cc(O)c2C(=O)O1	alpha-Zearalanol	1
C[C@H]1CCCC(O)CCC/C=C/c2cc(O)cc(O)c2C(=O)O1	alpha-Zearalenol	1
COc1cc(O)c2c(=O)oc3cc(O)cc(C)c3c2c1	alternariol monomethyl ether	1
CC(C)C1OC(=O)[C@H](Cc2ccccc2)N(C)C(=O)C(C(C)C)OC(=O)[C@H](Cc2ccccc2)N(C)C(=O)C(C(C)C)OC(=O)[C@H](Cc2ccccc2)N(C)C1=O	beauvericin	1
CC(C)[C@H]1OC(=O)[C@H](Cc2ccccc2)N(C)C(=O)[C@@H](C(C)C)OC(=O)[C@H](Cc2ccccc2)N(C)C(=O)[C@@H](C(C)C)OC(=O)[C@H](Cc2ccccc2)N(C)C1=O	beauvericin	1
CC(C)[C@@H]1OC(=O)[C@@H](Cc2ccccc2)N(C)C(=O)[C@H](C(C)C)OC(=O)[C@@H](Cc2ccccc2)N(C)C(=O)[C@H](C(C)C)OC(=O)[C@@H](Cc2ccccc2)N(C)C1=O	beauvericin	1
CC(C)C1OC(=O)C(Cc2ccccc2)N(C)C(=O)C(C(C)C)OC(=O)C(Cc2ccccc2)N(C)C(=O)C(C(C)C)OC(=O)C(Cc2ccccc2)N(C)C1=O	beauvericin	1
C[C@H]1CCC[C@@H](O)CCCCCc2cc(O)cc(O)c2C(=O)O1	beta-Zearalanol	1
C[C@H]1CCC[C@@H](O)CCC/C=C/c2cc(O)cc(O)c2C(=O)O1	beta-Zearalenol	1
CC1=C2C(=CO[C@H](C)[C@H]2C)C(=O)C(C(=O)O)=C1O	citrinin	1
CC1=C2C(=CO[C@@H](C)[C@@H]2C)C(=O)C(C(=O)O)=C1O	citrinin	1
Cc1c2oc3c(C)ccc(C(=O)N[C@@H]4C(=O)N[C@H](C(C)C)C(=O)N5CCC[C@H]5C(=O)N(C)CC(=O)N(C)[C@@H](C(C)C)C(=O)O[C@@H]4C)c3nc-2c(C(=O)N[C@@H]2C(=O)N[C@H](C(C)C)C(=O)N3CCC[C@H]3C(=O)N(C)CC(=O)N(C)[C@@H](C(C)C)C(=O)O[C@@H]2C)c(N)c1=O	dactinomycin	1
COc1cccc2c1C(=O)c1c(O)c3c(c(O)c1C2=O)C[C@@](O)(C(=O)CO)C[C@@H]3O[C@H]1C[C@H](N)[C@H](O)[C@H](C)O1	doxorubicin	1
Cc1cc(O)c2c(c1)C(=O)c1cc(O)cc(O)c1C2=O	emodin	1
CC[C@H](C)[C@H]1C(=O)OC(C(C)C)C(=O)N(C)[C@@H]([C@H](C)CC)C(=O)O[C@H](C(C)C)C(=O)N(C)[C@@H]([C@@H](C)CC)C(=O)O[C@H](C(C)C)C(=O)N1C	enniatins A	1
CC[C@H](C)[C@H]1C(=O)O[C@H](C(C)C)C(=O)N(C)[C@@H]([C@@H](C)CC)C(=O)O[C@H](C(C)C)C(=O)N(C)[C@@H](C(C)C)C(=O)O[C@H](C(C)C)C(=O)N1C	enniatin A1	1
CC(C)[C@H]1C(=O)O[C@H](C(C)C)C(=O)N(C)[C@@H](C(C)C)C(=O)O[C@H](C(C)C)C(=O)N(C)[C@@H](C(C)C)C(=O)O[C@H](C(C)C)C(=O)N1C	enniatin B	1
CC[C@H](C)[C@H]1C(=O)O[C@H](C(C)C)C(=O)N(C)[C@@H](C(C)C)C(=O)O[C@H](C(C)C)C(=O)N(C)[C@@H](C(C)C)C(=O)O[C@H](C(C)C)C(=O)N1C	enniatin B1	1
O=c1[nH]c(=O)n([C@H]2C[C@H](O)[C@@H](CO)O2)cc1F	floxuridine	1
CCCC[C@@H](C)[C@@H](OC(=O)C[C@@H](CC(=O)O)C(=O)O)[C@H](C[C@@H](C)CCCCCC[C@@H](O)C[C@H](O)[C@H](C)N)OC(=O)C[C@@H](CC(=O)O)C(=O)O	fumonisin B2	1
CCCCC(C)C(OC(=O)CC(CC(=O)O)C(=O)O)C(CC(C)CC(O)CCCCCCC(O)C(C)N)OC(=O)CC(CC(=O)O)C(=O)O	fumonisin B3	1
CCCCC(C)C(OC(=O)CC(CC(=O)O)C(=O)O)C(CC(C)CC(O)CCCCC(O)CC(O)C(C)N)OC(=O)CC(CC(=O)O)C(=O)O	fumonisin B	1
CO[C@@]12[C@H](COC(N)=O)C3=C(C(=O)C(C)=C(N)C3=O)N1C[C@@H]1N[C@@H]12	mitomycin C	1
O=c1cc(O)c1=O	monilformin	1
C[C@@H]1CC2=C(C=C(C(=C2C(=O)O1)O)C(=O)N[C@@H](CC3=CC=CC=C3)C(=O)O)Cl	ochratoxin A	1
O=C1C=C2C(=CCOC2O)O1	patulin	1
C=C(C)[C@@H]1NC(=O)[C@@H](NC)[C@@H](O)c2cc(Cl)c(O)c(c2)O[C@](C)(CC)[C@@H](C(=O)N2CC=C[C@H]2C(=O)N/C(C(=O)N/C(=C/C(=O)O)C(=O)O)=C(\C)CC)NC1=O	phomopsin A	1
COC(=O)C12Oc3ccc(-c4ccc5c(c4O)C(O)=C4C(=O)CC(C)C(O)C4(C(=O)OC)O5)c(O)c3C(O)=C1C(=O)CC(C)C2O	secalonic acid D	1
COc1c(Cl)cc2c(c1OC)N(C)[C@@H]1N3C(=O)[C@@]4(C)SS[C@@]3(C(=O)N4C)[C@H](O)[C@]21O	sporidesmin	1
COc1cc2c(c3oc4cccc(O)c4c(=O)c13)[C@@H]1C=CO[C@@H]1O2	sterigmatocystin	1
CC1=CC2OC3[C@H](O)C[C@@](C)(C34CO4)[C@@]2(CO)[C@H](O)C1=O	vomitoxin	1
C[C@H]1CCCC(=O)CCCCCc2cc(O)cc(O)c2C(=O)O1	zearalanone	1
C[C@H]1CCCC(=O)CCC/C=C/c2cc(O)cc(O)c2C(=O)O1	zearalenone	1
C[C@H]1CCCC(O)CCC/C=C/c2cc(O)cc(O)c2C(=O)O1	alpha-zearalenol	1
C[C@H]1CCC[C@H](O)CCC/C=C/c2cc(O)cc(O)c2C(=O)O1	alpha-zearalenol	1
C[C@H]1CCC[C@@H](O)CCC/C=C/c2cc(O)cc(O)c2C(=O)O1	beta-zearalenol	1
C[C@H]1CCC[C@H](O)CCCCCc2cc(O)cc(O)c2C(=O)O1	zeranol	1

**Table A10:** Validation set for carcinogenicity data.

SMILES	Name	y
CCCCOC(=O)c1ccc(O)cc1	butylparaben	0
O=C(NC(CO)C(O)c1ccc([N+](=O)[O−])cc1)C(Cl)Cl	bhloramphenicol	0
O=C(N[C@H](CO)[C@H](O)c1ccc([N+](=O)[O−])cc1)C(Cl)Cl	chloramphenicol	0
O=C(N[C@@H](CO)[C@@H](O)c1ccc([N+](=O)[O−])cc1)C(Cl)Cl	chloramphenicol	0
O=C(N[C@@H](CO)[C@H](O)c1ccc([N+](=O)[O−])cc1)C(Cl)Cl	chloramphenicol	0
O=C(N[C@H](CO)[C@@H](O)c1ccc([N+](=O)[O−])cc1)C(Cl)Cl	chloramphenicol	0
O=C(N[C@H](CO)[C@H](O)[13c]1[13cH][13cH][13c]([N+](=O)[O−])[13cH][13cH]1)C(Cl)Cl	chloramphenicol	0
[2H]c1c([2H])c([C@@]([2H])(O)[C@@H](CO)NC(=O)C(Cl)Cl)c([2H])c([2H])c1[N+](=O)[O−]	chloramphenicol	0
O=[13C](N[13C@H]([13CH2]O)[13C@H](O)[13c]1ccc([N+](=O)[O−])cc1)[13CH](Cl)Cl	chloramphenicol	0
[2H]C(Cl)(Cl)C(=O)N[C@]([2H])(C([2H])([2H])O)[C@]([2H])(O)c1ccc([N+](=O)[O−])cc1	chloramphenicol	0
[2H]c1c([2H])c(C([2H])(O)C(CO)NC(=O)C(Cl)Cl)c([2H])c([2H])c1[N+](=O)[O−]	chloramphenicol	0
[3H]c1cc([C@@H](O)[C@@H](CO)NC(=O)C(Cl)Cl)cc([3H])c1[N+](=O)[O−]	chloramphenicol	0
[2H]c1c([2H])c([C@@]([2H])(O)[C@H](CO)NC(=O)C(Cl)Cl)c([2H])c([2H])c1[N+](=O)[O−]	chloramphenicol	0
O=[13C](N[13C@H]([13CH2]O)[13C@H](O)[13c]1[13cH][13cH][13c]([N+](=O)[O−])[13cH][13cH]1)[13CH](Cl)Cl	chloramphenicol	0
[2H]OC[C@H]([C@H](O[2H])c1ccc([N+](=O)[O−])cc1)N([2H])C(=O)C(Cl)Cl	chloramphenicol	0
C/C=C/C[C@@H](C)[C@@H](O)[C@H]1C(=O)N[C@@H](CC)C(=O)N(C)CC(=O)N(C)[C@@H](CC(C)C)C(=O)N[C@@H](C(C)C)C(=O)N(C)[C@@H](CC(C)C)C(=O)N[C@@H](C)C(=O)N[C@H](C)C(=O)N(C)[C@@H](CC(C)C)C(=O)N(C)[C@@H](CC(C)C)C(=O)N(C)[C@@H](C(C)C)C(=O)N1C	cyclosporin A	0
CC1=C[C@H]2O[C@@H]3[C@H](O)C[C@@](C)([C@]34CO4)[C@@]2(CO)[C@H](O)C1=O	deoxynivalenol	0
[2H]c1c(C([2H])([2H])[2H])cc(O)c2c1C(=O)c1cc(O)cc(O)c1C2=O	emodin	0
CC(=O)O[C@@H]1[C@@H](O)[C@H]2O[C@@H]3C=C(C)C(=O)[C@@H](O)[C@]3(CO)[C@]1(C)[C@]21CO1	fusarenon X	0
CC(C)COC(=O)c1ccc(O)cc1	isobutyl 4-hydroxybenzoate	0
CO[C@@H]1[C@@H](O[C@@H]2O[C@H](C)[C@@H](O[C@H]3C[C@@](C)(O)[C@@H](OC(=O)CC(C)C)[C@H](C)O3)[C@H](N(C)C)[C@H]2O)[C@@H](CC=O)C[C@@H](C)[C@@H](O)/C=C/C=C/C[C@@H](C)OC(=O)C[C@H]1OC(C)=O	josamycin	0
COC(C(=O)O)c1ccccc1	methoxyphenylacetic acid	0
CC1=C[C@H]2O[C@@H]3[C@H](O)[C@@H](O)[C@@](C)([C@]34CO4)[C@@]2(CO)[C@H](O)C1=O	nivalenol	0
CN(C)[C@@H]1C(=O)C(C(N)=O)=C(O)[C@@]2(O)C(=O)C3=C(O)c4c(O)cccc4[C@@](C)(O)[C@H]3[C@H](O)[C@@H]12.Cl	oxytetracycline	0
O=[13C]1[13CH]=[13C]2[13C](=[13CH][13CH2]O[13CH]2O)O1	patulin	0
CN(C)[C@@H]1C(=O)C(C(N)=O)=C(O)[C@@]2(O)C(=O)C3=C(O)c4c(O)cccc4[C@@](C)(O)[C@H]3C[C@@H]12.Cl	tetracycline	0
Oc1cc(Cl)ccc1Oc1ccc(Cl)cc1Cl	triclosan	0
CC1=C[C@@H]2[C@]([C@@H](C1=O)O)([C@]3(C[C@H]([C@H]([C@]34CO4)O2)O)C)CO	vomitoxin	0
[2H]C([2H])([2H])Oc1cc2c(c3oc(=O)c4c(c13)CCC4=O)[C@@H]1CCO[C@@H]1O2	1217830-52-8	1
C[C@H]1Cc2c(Cl)cc(C(=O)N[C@@H](Cc3ccccc3)C(=O)O)c(O)c2C(=O)O1	3-epi-Ochratoxin A	1
C[C@H]1Cc2c(Cl)cc(C(=O)N[C@H](Cc3ccccc3)C(=O)O)c(O)c2C(=O)O1	3S14R-Ochratoxin A	1
O=c1[nH]c(=O)n([C@H]2C[C@H](O)[C@@H](CO)O2)cc1Br	5-BROMO-2′-DEOXYURIDINE	1
Cc1c2oc3c(C)ccc(C(=O)NC4C(=O)NC(C(C)C)C(=O)N5CCC[C@H]5C(=O)N(C)CC(=O)N(C)C(C(C)C)C(=O)OC4C)c3nc-2c(C(=O)NC2C(=O)NC(C(C)C)C(=O)N3CCCC3C(=O)N(C)CC(=O)N(C)C(C(C)C)C(=O)OC2C)c(N)c1=O	A-2600	1
Cc1c2oc3c(C)ccc(C(=O)NC4C(=O)N[C@H](C(C)C)C(=O)N5CCC[C@H]5C(=O)N(C)CC(=O)N(C)[C@@H](C(C)C)C(=O)O[C@@H]4C)c3nc-2c(C(=O)NC2C(=O)N[C@H](C(C)C)C(=O)N3CCC[C@H]3C(=O)N(C)CC(=O)N(C)[C@@H](C(C)C)C(=O)O[C@@H]2C)c(N)c1=O	actinomycin D	1
Cc1c2oc3c(C)ccc(C(=O)NC4C(=O)NC(C(C)C)C(=O)N5CCCC5C(=O)N(C)CC(=O)N(C)C(C(C)C)C(=O)OC4C)c3nc-2c(C(=O)NC2C(=O)NC(C(C)C)C(=O)N3CCCC3C(=O)N(C)CC(=O)N(C)C(C(C)C)C(=O)OC2C)c(N)c1=O	actinomycin D	1
Cc1c2oc3c(C)ccc(C(=O)N[C@@H]4C(=O)N[C@H](C(C)C)C(=O)N5CCC[C@H]5C(=O)N(C)CC(=O)N(C)[C@@H](C(C)C)C(=O)O[C@H]4C)c3nc-2c(C(=O)N[C@@H]2C(=O)N[C@H](C(C)C)C(=O)N3CCC[C@H]3C(=O)N(C)CC(=O)N(C)[C@@H](C(C)C)C(=O)O[C@H]2C)c(N)c1=O	actinomycin D	1
Cc1c2oc3c(C)ccc(C(=O)N[C@@H]4C(=O)N[C@H](C(C)C)C(=O)N5CCC[C@H]5C(=O)N(C)CC(=O)N(C)[C@H](C(C)C)C(=O)O[C@@H]4C)c3nc-2c(C(=O)N[C@@H]2C(=O)N[C@H](C(C)C)C(=O)N3CCC[C@H]3C(=O)N(C)CC(=O)N(C)[C@@H](C(C)C)C(=O)O[C@@H]2C)c(N)c1=O	actinomycin D	1
Cc1c2oc3c(C)ccc(C(=O)N[C@@H]4C(=O)N[C@@H](C(C)C)C(=O)N5CCC[C@H]5C(=O)N(C)CC(=O)N(C)[C@@H](C(C)C)C(=O)O[C@@H]4C)c3nc-2c(C(=O)N[C@@H]2C(=O)N[C@@H](C(C)C)C(=O)N3CCC[C@H]3C(=O)N(C)CC(=O)N(C)[C@@H](C(C)C)C(=O)O[C@@H]2C)c(N)c1=O	actinomycin	1
[3H]c1c([3H])c(C([3H])([3H])[3H])c2oc3c(C([3H])([3H])[3H])c(=O)c(N([3H])[3H])c(C(=O)N([3H])[C@]4([3H])C(=O)N([3H])[C@]([3H])(C([3H])(C([3H])([3H])[3H])C([3H])([3H])[3H])C(=O)N5C([3H])([3H])C([3H])([3H])C([3H])([3H])[C@]5([3H])C(=O)N(C([3H])([3H])[3H])C([3H])([3H])C(=O)N(C([3H])([3H])[3H])[C@@]([3H])(C([3H])(C([3H])([3H])[3H])C([3H])([3H])[3H])C(=O)O[C@]4([3H])C([3H])([3H])[3H])c-3nc2c1C(=O)N([3H])[C@]1([3H])C(=O)N([3H])[C@]([3H])(C([3H])(C([3H])([3H])[3H])C([3H])([3H])[3H])C(=O)N2C([3H])([3H])C([3H])([3H])C([3H])([3H])[C@]2([3H])C(=O)N(C([3H])([3H])[3H])C([3H])([3H])C(=O)N(C([3H])([3H])[3H])[C@@]([3H])(C([3H])(C([3H])([3H])[3H])C([3H])([3H])[3H])C(=O)O[C@]1([3H])C([3H])([3H])[3H]	actinomycin D	1
Cc1c2oc3c(C)ccc(C(=O)N[C@@H]4C(=O)N[C@H](C(C)C)C(=O)N5CCC[C@H]5C(=O)N(C)CC(=O)N(C)[C@@H](C(C)C)C(=O)O[C@@H]4C)c3nc-2c(C(=O)N[C@H]2C(=O)N[C@H](C(C)C)C(=O)N3CCC[C@@H]3C(=O)N(C)CC(=O)N(C)[C@@H](C(C)C)C(=O)O[C@@H]2C)c(N)c1=O	actinomycin D	1
Cc1c2oc3c(C)ccc(C(=O)N[C@@H]4C(=O)N[C@H](C(C)C)C(=O)N5CCC[C@H]5C(=O)N(C)CC(=O)N(C)C(C(C)C)C(=O)O[C@@H]4C)c3nc-2c(C(=O)N[C@@H]2C(=O)N[C@H](C(C)C)C(=O)N3CCC[C@H]3C(=O)N(C)CC(=O)N(C)[C@@H](C(C)C)C(=O)O[C@@H]2C)c(N)c1=O	actinomycin D	1
COc1cc2c(c3oc(=O)c4c(c13)CCC4=O)C1C=COC1O2	aflatoxin BI	1
COc1cc2c(c3oc(=O)c4c(c13)CC[C@@H]4O)[C@@H]1C=CO[C@@H]1O2	aflatoxicol	1
COc1cc2c(c3oc(=O)c4c(c13)CCC4=O)[C@@H]1C=CO[C@@H]1O2	aflatoxin B1	1
[13CH3]O[13c]1[13cH][13c]2[13c]([13c]3o[13c](=O)[13c]4[13c]([13c]13)[13CH2][13CH2][13C]4=O)[13C@@H]1[13CH]=[13CH]O[13C@@H]1O2	aflatoxin B1-13C17	1
COc1cc2c(c3oc(=O)c4c(c13)CCC4=O)[C@@H]1CCO[C@@H]1O2	aflatoxin B2	1
COc1cc2c(c3oc(=O)c4c(c13)CCC4=O)C1CCOC1O2	aflatoxin B2 alpha	1
COc1cc2c(c3oc(=O)c4c(c13)CCOC4=O)C1C=COC1O2	aflatoxin G1	1
COc1cc2c(c3oc(=O)c4c(c13)CCOC4=O)[C@@H]1C=CO[C@@H]1O2	aflatoxin G1	1
COc1cc2c(c3oc(=O)c4c(c13)CCOC4=O)[C@H]1C=CO[C@@H]1O2	aflatoxin G1	1
COc1cc2c(c3oc(=O)c4c(c13)CCOC4=O)[C@H]1C=CO[C@H]1O2	aflatoxin G1	1
[13CH3]O[13c]1[13cH][13c]2[13c]([13c]3o[13c](=O)[13c]4[13c]([13c]13)[13CH2][13CH2]O[13C]4=O)[13C@@H]1[13CH]=[13CH]O[13C@@H]1O2	aflatoxin G1-13C17	1
COc1cc2c(c3oc(=O)c4c(c13)CCOC4=O)[C@@H]1CCO[C@@H]1O2	aflatoxin G2	1
[13CH3]O[13c]1[13cH][13c]2[13c]([13c]3o[13c](=O)[13c]4[13c]([13c]13)[13CH2][13CH2]O[13C]4=O)[13C@@H]1[13CH2][13CH2]O[13C@@H]1O2	aflatoxin G2-13C17	1
CC1Cc2c(Cl)cc(C(=O)NC(Cc3ccccc3)C(=O)O)c(O)c2C(=O)O1	antibiotic 9663	1
COc1cc2c(c3oc4cccc(O)c4c(=O)c13)C1C=CO[C@@H]1O2	CHEMBL1532401	1
CC1=C2C(=CO[C@H](C)[C@H]2C)C(=O)C(C(=O)O)=C1O	citrinin	1
CC[C@@H]1NC(=O)[C@@H]2[C@H](Cl)[C@H](Cl)CN2C(=O)[C@H](CO)NC(=O)C[C@H](c2ccccc2)NC(=O)[C@H](CO)NC1=O	cyclochlorotine	1
Cc1c2oc3c(C)ccc(C(=O)N[C@@H]4C(=O)N[C@H](C(C)C)C(=O)N5CCC[C@H]5C(=O)N(C)CC(=O)N(C)[C@@H](C(C)C)C(=O)O[C@@H]4C)c3nc-2c(C(=O)N[C@@H]2C(=O)N[C@H](C(C)C)C(=O)N3CCC[C@H]3C(=O)N(C)CC(=O)N(C)[C@@H](C(C)C)C(=O)O[C@@H]2C)c(N)c1=O	dactinomycin	1
COc1cccc2c1C(=O)c1c(O)c3c(c(O)c1C2=O)C[C@@](O)(C(C)=O)C[C@@H]3O[C@H]1C[C@H](N)[C@H](O)[C@H](C)O1	daunorubicin	1
COc1cc2c(c3oc(=O)c4c(c13)CCOC4=O)C1CCOC1O2	dihydroaflatoxin G1	1
COc1cccc2c1C(=O)c1c(O)c3c(c(O)c1C2=O)C[C@@](O)(C(=O)CO)C[C@@H]3O[C@H]1C[C@H](N)[C@H](O)[C@H](C)O1	doxorubicin	1
C=C1C[C@]23C[C@@]1(O)CC[C@H]2[C@@]12C=C[C@H](O)[C@@](C)(C(=O)O1)[C@H]2[C@@H]3C(=O)O	gibberellic acid	1
COC1=CC(=O)C[C@@H](C)[C@]12Oc1c(Cl)c(OC)cc(OC)c1C2=O	griseofulvin	1
CC[C@H](C)C(=O)O[C@H]1C[C@H](C=C2[C@H]1[C@H]([C@H](C=C2)C)CC[C@@H]3C[C@H](CC(=O)O3)O)C	lovastatin	1
Cc1cc(O)c2c(c1O)C(=O)C13C(=C2O)C(=O)C2C(O)C1C1C(O)C3C(=O)C3=C(O)c4c(O)cc(C)c(O)c4C(=O)C321	luteoskyrin	1
Cc1cc(O)c2c(c1O)C(=O)[C@]13C(=C2O)C(=O)[C@@H]2[C@H](O)[C@H]1[C@@H]1[C@@H](O)[C@H]3C(=O)C3=C(O)c4c(O)cc(C)c(O)c4C(=O)[C@@]321	luteoskyrin	1
C=CCc1ccc(OC)c(OC)c1	methyl eugenol	1
CO[C@@]12[C@H](COC(N)=O)C3=C(C(=O)C(C)=C(N)C3=O)N1C[C@@H]1N[C@@H]12	mitomycin C	1
C[C@@H]1Cc2c(Cl)cc(C(=O)N[C@H](Cc3ccccc3)C(=O)O)c(O)c2C(=O)O1	N-[(3R)-5-chloro-8-hydroxy-3-methyl-1-oxo-3,4-dihydro-1H-2-benzopyran-7-carbonyl]-D-phenylalanine	1
C[C@@H]1Cc2c(Cl)cc(C(=O)N[C@@H](Cc3ccccc3)C(=O)O)c(O)c2C(=O)O1	ochratoxin A	1
CC1Cc2c(Cl)cc(C(=O)N[C@H](Cc3ccccc3)C(=O)O)c(O)c2C(=O)O1	ochratoxin-A	1
CC/C(=C(\C(=O)N/C(=C/C(=O)O)/C(=O)O)/NC(=O)[C@@H]1C=CCN1C(=O)[C@@H]2[C@@](OC3=C(C(=CC(=C3)[C@@H]([C@@H](C(=O)N[C@H](C(=O)N2)C(=C)C)NC)O)Cl)O)(C)CC)/C	phomopsin A	1
COc1cc2c(c3oc4cccc(O)c4c(=O)c13)[C@@H]1C=CO[C@@H]1O2	sterigmatocystin	1
COc1cc2c(c3oc4cccc(O)c4c(=O)c13)C1C=COC1O2	sterigmatocystin	1

**Table A11:** Prediction metrics of the external validation set of mycotoxins with 4 different QSAR prediction tools.

Model	Accuracy	Sensitivity	Specificity	Precision	f1	TN	FP	FN	TP	Num of Cpds
**Carcinogenicity ProtoPRED**	**0.80**	**0.74**	**0.89**	**0.92**	**0.82**	**25**	**3**	**12**	**35**	**75**
Carcinogenicity QTB Case Ultra	0.71	0.75	0.65	0.77	0.76	15	8	9	27	59 *
Carcinogenicity QTB Leadscope	0.59	0.62	0.53	0.65	0.63	9	8	9	15	41 *
Carcinogenicity VEGA	0.70	0.56	0.81	0.67	0.61	21	5	8	10	31 **
**In vitro genotoxicity mycotoxin model**	**0.93**	**1.00**	**0.86**	**1.00**	**0.94**	**6**	**0**	**1**	**8**	**15**
In vitro genotoxicity VEGA	0.54	1.00	0.00	0.54	0.70	0	6	0	7	13 **
**In vivo genotoxicity ProtoPRED**	**0.81**	**0.88**	**0.46**	**0.88**	**0.88**	**6**	**7**	**7**	**52**	**72**
In vivo genotoxicity QTB Case Ultra	0.62	0.60	0.67	0.75	0.67	2	1	2	3	8 *
In vivo genotoxicity QTB Leadscope	0.65	0.64	0.67	0.82	0.72	4	2	5	9	20 *
In vivo genotoxicity VEGA	0.39	0.33	0.57	0.70	0.45	4	3	14	7	28 ***
**Mutagenicity mycotoxin model**	**0.83**	**0.92**	**0.75**	**0.92**	**0.85**	**9**	**1**	**3**	**11**	**24**
Mutagenicity QTB Case Ultra	0.94	0.90	1.00	1.00	0.95	8	0	1	9	18 *
Mutagenicity QTB Leadscope	0.88	0.86	0.90	0.86	0.86	9	1	1	6	17 *
Mutagenicity VEGA	0.67	0.83	0.50	0.62	0.71	6	6	2	10	24

QTB = QSARToolbox; FN = false negatives; TN = true positives; FP = false positives; TP = true positives. The models described in this article are labelled in bold. * Several compounds could not be predicted because they were not included in the applicability domain of the model. ** Some compounds could not be predicted because the program did not accept the input structure. *** Some compounds could not be predicted because the program did not accept the input structure or the prediction was unclear.
